# Post-hepatectomy liver regeneration in the context of bile acid homeostasis and the gut-liver signaling axis

**DOI:** 10.18053/jctres.04.201801.001

**Published:** 2018-02-16

**Authors:** Lianne de Haan, Sarah J. van der Lely, Anne-Loes K. Warps, Quincy Hofsink, Pim B. Olthof, Mark J. de Keijzer, Daniël A. Lionarons, Lionel Mendes-Dias, Bote G. Bruinsma, Korkut Uygun, Hartmut Jaeschke, Geoffrey C. Farrell, Narci Teoh, Rowan F. van Golen, Tiangang Li, Michal Heger

**Affiliations:** ^1^ *Department of Experimental Surgery, Academic Medical Center, University of Amsterdam, Amsterdam, the Netherlands*; ^2^ *Oncogene Biology Laboratory, Francis Crick Institute, London, United Kingdom*; ^3^ *Center for Engineering in Medicine, Department of Surgery, Massachusetts General Hospital, Harvard Medical School, Boston, MA, United States*; ^4^ *Shriners Hospitals for Children, Boston, MA, United States*; ^5^ *Department of Pharmacology, Toxicology and Therapeutics, University of Kansas Medical Center, KS, United States*; ^6^ *Liver Research Group, Australian National University Medical School at the Canberra Hospital, Canberra, Australian Capital Territory, Australia*; ^7^ *Membrane Biochemistry and Biophysics, Bijvoet Center for Biomolecular Research, Institute of Biomembranes, Utrecht University, Utrecht, the Netherlands*; ^#^ *Present address: Department of Surgery, Reinier de Graaf Gasthuis, Delft, the Netherlands*

**Keywords:** surgery, mitotic signaling, hepatocyte proliferation, arnesoid X receptor enteral, fibroblast growth factor, detoxification, transport and canalicular excretion

## Abstract

**Background:**

Liver regeneration following partial hepatectomy (PHx) is a complicated process involving multiple organs and several types of signaling networks. The bile acid-activated metabolic pathways occupy an auxiliary yet important chapter in the entire biochemical story. PHx is characterized by rapid but transient bile acid overload in the liver, which constitutes the first wave of proliferative signaling in the remnant hepatocytes. Bile acids trigger hepatocyte proliferation through activation of several nuclear receptors. Following biliary passage into the intestines, enterocytes reabsorb the bile acids, which results in the activation of farnesoid X receptor (FXR), the consequent excretion of fibroblast growth factor (FGF)19/FGF15, and its release into the enterohepatic circulation. FGF19/FGF15 subsequently binds to its cognate receptor, fibroblast growth factor receptor 4 (FGFR4) complexed with β-klotho, on the hepatocyte membrane, which initiates the second wave of proliferative signaling. Because some bile acids are toxic, the remnant hepatocytes must resolve the potentially detrimental state of bile acid excess. Therefore, the hepatocytes orchestrate a bile acid detoxification and elimination response as a protective mechanism in concurrence with the proliferative signaling. The response in part results in the excretion of (biotransformed) bile acids into the canalicular system, causing the bile acids to end up in the intestines.

**Relevance for patients:**

Recently, FXR agonists have been shown to promote regeneration via the gut-liver axis. This type of pharmacological intervention may prove beneficial for patients with hepatobiliary tumors undergoing PHx. In light of these developments, the review provides an in-depth account of the pathways that underlie post-PHx liver regeneration in the context of bile acid homeostasis in the liver and the gut-liver signaling axis.

## 1. Introduction

The liver strictly maintains its size at a predefined setpoint in order to optimally fulfill its detoxification-, synthesis-, immunological-, and endocrinological functions [1]. Under pathological conditions, the host is not only able to sense a loss of viable liver tissue, but also to mount a regenerative response so as to rapidly restore original liver size and function. Factors that control the liver-to-body weight ratio, or the ‘hepatostat,’ are only partially understood [2].

The prototypical stimulus for liver regeneration is the surgical removal of part of the organ (partial hepatectomy, PHx), as is routinely performed by surgeons most often in case of hepatobiliary malignancies [3]. Rodent PHx models have been extensively employed to study the mechanisms that underlie post-PHx liver regeneration. Owing to these models we now know that, immediately after PHx, the activation of early response transcription factors [4,5] and mitogen-activated protein kinases (MAPKs) [6] leads to hepatocyte proliferation and compensatory liver regrowth. These growth signals are activated directly after PHx by hemodynamic changes [7], inflammation [8-10], and cell damage [11-14]. Proliferation is perpetuated until the liver size reaches a mass that complies with the hepatostat, at which point liver growth is terminated [15,16].

The regenerative capacity of the liver after PHx is not inexhaustible. As a reduced number of hepatocytes have to uphold all metabolic functions whilst the liver reclaims its original size, there is a risk of developing liver failure if the liver remnant is too small or too frail [17], a condition which is often referred to as small-for-size syndrome [ 18]. Several sensors involved in hemodynamic changes [7], inflammation [8-10], cell damage [11-14], and bile acid metabolism [19,20] are embedded to foster successful liver regeneration and prevent liver failure [21]. Sensors that are involved in bile acid metabolism are especially important in proregenerative signaling through the gut-liver axis [22,25]. Bile acid receptors such as farnesoid X receptor (FXR) promote liver regeneration and prevent liver failure by (i) modulating the bile acid pool size, composition, and compartmentalization [21,26], (ii) governing the production of mitogens such as fibroblast growth factor 15/19 (FGF15/19, signifying rodent/human orthologues) [22,23], and (iii) rewiring mitochondrial metabolism to fuel liver growth [27]. In addition to coordinating liver regeneration after PHx, bile acids are also able to override the hepatostat and consequently expand liver size to larger than normal without concurrent mitogenic triggers [28,29], underscoring their prolific role in compensatory liver regrowth.

As (semi-)synthetic selective agonists of bile acid- and other nuclear receptors have become available [30-33], the metabolic components of liver regeneration could be exploited to pharmacologically enhance liver growth, which in turn could benefit numerous medical scenarios. This review therefore summarizes the molecular pathways that lie at the basis of post-hepatectomy liver regeneration in the context of bile acid homeostasis and the gut-liver signaling axis.

## 2. General mechanisms of liver regeneration following partial hepatectomy

PHx-induced liver regeneration involves all cell types in the liver, including hepatocytes, Kupffer cells, stellate cells, biliary epithelial cells, and endothelial cells [34]. The time lapse of proliferation is distinct for every cell type. In the rat liver, hepatocytes begin DNA synthesis at 12 hours after PHx, which peaks at 24 hours. The onset of hepatocellular DNA synthesis is initiated in the vicinity of the portal veins and subsequently spreads towards the central veins [35]. The peak of DNA synthesis in non-parenchymal cells is later. Kupffer cells start to proliferate at 48 hours, and biliary epithelial cells and endothelial cells at 96 hours after PHx. The most profound increase in liver mass in rats occurs during the first 3 days after PHx, and restoration of the remnant liver to its original liver mass is completed within 7-14 days [24,36,37]. In humans, recovery of pre-operative liver function takes place within the first 10 days after PHx [38], but complete regrowth of the remnant human liver occurs 3-6 months after PHx [39]. Although all cell types contribute to the increase in liver mass after PHx, this review will primarily focus on the cell cycle progression and proliferation of hepatocytes.

Liver regeneration entails the activation of multiple regulatory pathways that include cytokine-, growth factor-, and metabolic networks [40]. More specifically, PHx induces differential regulation of genes that coordinate cell cycle regulation, chromatin reorganization, transcriptional regulation, signal transduction, protein targeting, metabolism, transport, xenobiotic metabolism, surface receptors, inflammation, and acute phase responses [41]. These pathways are well-coordinated to allow proper restoration of the tissue while maintaining vital liver functions. A global overview is provided in the next subsections. For more detailed information, interested readers are referred to specialized publications [36,37,42,43].

### 2.1 Initial triggers of liver regeneration: changes in hepatic hemodynamics, sterile and non-sterile inflammation, and a shift in intracellular redox state

**Figure 1.**
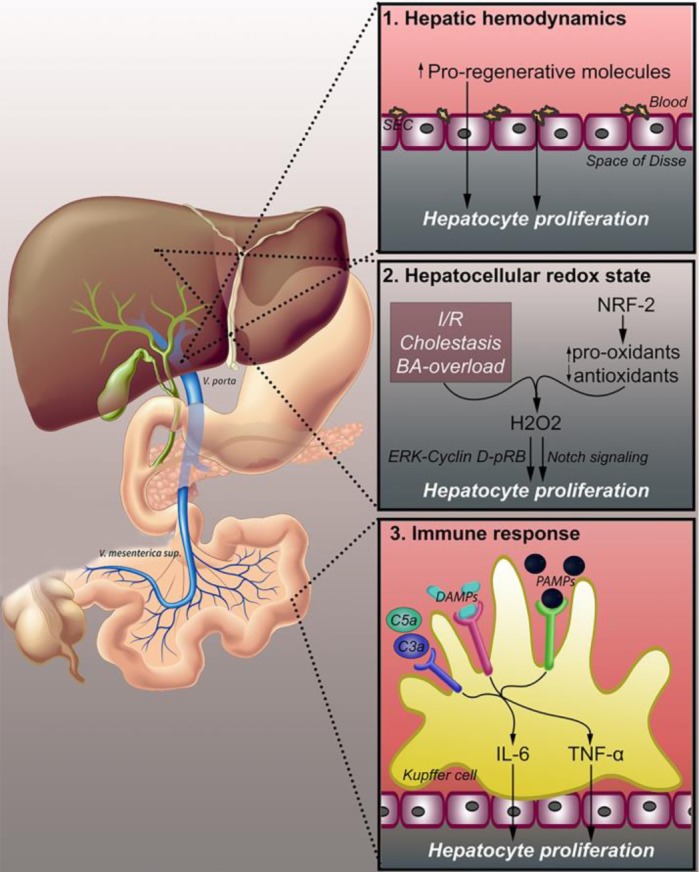
Changes in hepatic hemodynamics that lead to liver regeneration. Three physiological changes following PHx trigger liver regeneration. Altered hepatic hemodynamics (**1**) lead to increased hepatic exposure to pro-regenerative factors originating from the portal circulation. Additionally, platelets accumulate in the space of Disse and release pro-regenerative molecules. The hepatocellular redox state (**2**) shifts to a pro-oxidative state due to ischemia/reperfusion, cholestasis, and bile acid overload. NRF-2 and other redox-active enzymes upregulate pro-oxidant enzymes and downregulates antioxidant enzymes, leading to increased levels of H2O2, which promotes cell proliferation through both ERK-cyclin D1-p-RB and Notch signaling. PHx induces hepatocyte proliferation through an immune response (**3**), resulting from endotoxemia, intestine-derived PAMPs, and damaged cells leaking DAMPs. PAMPs and DAMPs bind PRRs on Kupffer cells, triggering the release of cytokines such as TNF-α and IL-6. Complement factors C3a and C5a are also triggered by the immune response and activate TNF-α and IL-6 release through complement receptors. Abbreviations: SEC, sinusoidal endothelial cell; BA, bile acid; NRF-2, nuclear factor (erythroid-derived 2)-like 2; H2O2, hydrogen peroxide; ERK, extracellular signal-regulated kinase; pRB, phosphorylated retinoblastoma protein; DAMPs, damage-associated molecular patterns; PRR, pattern recognition receptor; PAMPs, pathogen-associated molecular patterns; IL-6, interleukin 6; TNF-α, tumor necrosis factor alpha.

The first physiological change during PHx is the redirection of portal and arterial blood supply to the remnant liver instead of the entire liver [44]. As a result, hepatocytes become exposed to a 3-fold greater amount of proregenerative factors [44], mainly supplied by the portal vein (Figure 1). The post-PHx hemodynamic heterogeneity [45-47] and portal hypertension [25] facilitate platelet-endothelial cell interactions during both stasis and flow [48,49]. Moreover, the fenestrations between the sinusoidal endothelial cells widen after PHx [50], allowing more facile passage of blood-borne signaling molecules but also platelets into the space of Disse [51,52].

**Figure 2.**
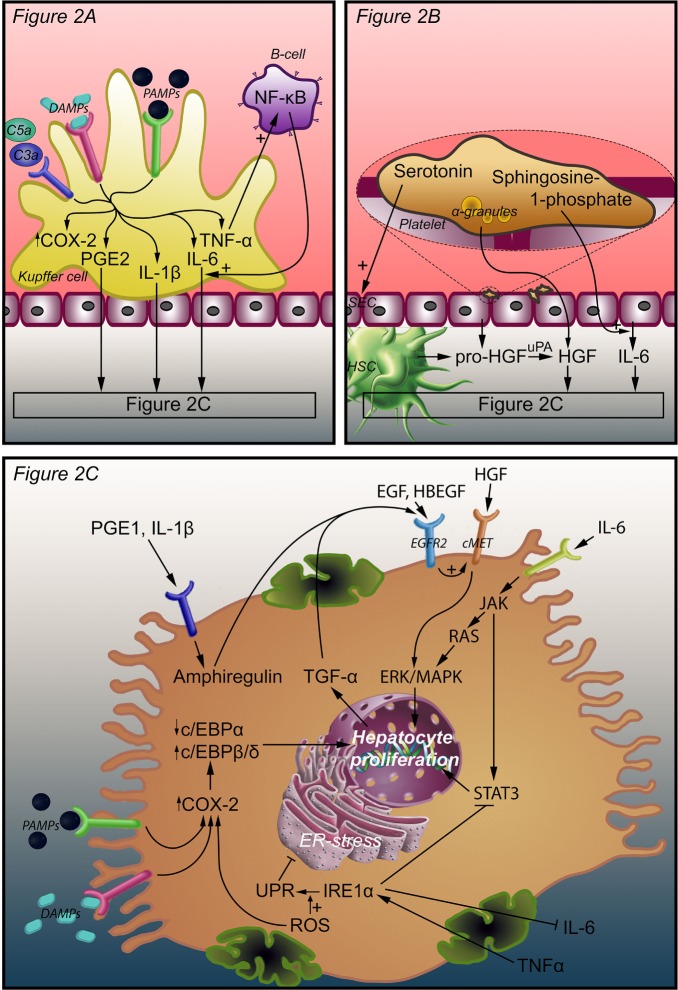
Intercellular and intracellular signals that initiate liver regeneration. Three mechanisms lead to hepatocyte proliferation. In response to complement factors C3a, C5a, PAMPs, and DAMPs, Kupffer cells release TNF-α, IL-6, PGE2, and IL-1β. TNF-α induces an autocrine loop through NF-κB production by B-cells. IL-6, PGE2, and IL-1β bind their cognate receptors on hepatocytes (**A**). Platelets accumulate in the space of Disse and release an armament of growth factors, including HGF, from their α-granules. Pro-HGF is released from SECs and HSCs and is converted to HGF by uPA, which is activated as a result of ECM damage after PHx. Platelets also release serotonin, which stimulates SECs, and sphringosine-1-phosphate to stimulate IL-6 release (**B**). PGE2 and IL-1β coming from Kupffer cells stimulate amphiregulin production in hepatocytes. HGF binds its receptor c-Met and activates hepatocyte proliferation through ERK-1/2 MAPK. IL-6 binds the IL-6 receptor and activates JAK, which induces hepatocyte proliferation through STAT3 and through the RAS-ERK-1/2 MAPK pathway. EGF, TGF-α, HBEGF, and amphiregulin enhance the effect of HGF through EGFR2. EGF is produced in the duodenum and reaches the liver through the portal circulation. TGF-α is produced by proliferating hepatocytes. HBEGF is released from monocytes and macrophages and converted to its active form by metalloproteinases. TNF-α (from Kupffer cells) and ROS enhance the UPR that is mediated by IRE1α. The UPR inhibits ER stress that in turn activates COX-2 expression. COX-2 is also stimulated by ROS, PAMPs, and DAMPs. Enhanced COX-2 expression increases C/EBPβ and C/EBPδ expression and decreases C/EBPα expression, thereby stimulating hepatocyte proliferation (C). Abbreviations: DAMPs, damage-associated molecular patterns; PAMPs, pathogen-associated molecular patterns; NF-κB, nuclear factor kappa-light-chain-enhancer of activated B-cells; COX-2, cyclooxygenase 2; PGE2, prostaglandin E2; IL-1β, interleukin 1β; IL-6, interleukin 6; TNF-α, tumor necrosis factor alpha; SEC, sinusoidal endothelial cell; HSC, hepatic stellate cell; HGF, hepatocyte growth factor; uPA, urokinase plasminogen activator; C/EBPα, β, and δ, CCAAT enhancer binding protein alpha, beta, and delta; TGF-α, tumor growth factor alpha; EGF, epidermal growth factor; EGFR2, epidermal growth factor receptor 2; JAK, Janus kinase; ERK, extracellular signal-regulated kinase; MAPK, mitogen-activated protein kinase; STAT3, signal transducer and activator of transcription 3; IRE1α, inositol-requiring enzyme-1α; UPR, unfolded protein response; ROS, reactive oxygen species.

Secondly, the surgical trauma after PHx causes damaged and dying cells to leak their intracellular content [11,12], which contains damage-associated molecular patterns (DAMPs), into the extracellular compartment. The DAMPs bind pattern recognition receptors (PRR) such as Toll-like receptors (TLRs) on Kupffer cells and trigger a sterile immune response [13,14], characterized by the release of tumor necrosis factor-α (TNF-α) and interleukin-6 (IL-6) from Kupffer cells [8], as illustrated in Figure 1 and Figure 2. These cytokines trigger proliferative signaling in hepatocytes and subsequent liver regeneration [36,53-55]. PHx-induced injury also triggers the complement peptides C3a (mice and humans) and C5a (mice) complement activation [8-10], which bind to complement receptors on Kupffer cells and neutrophils and amplify the sterile immune response [8-10]. The consequent immune cell activation, cytokine production, and release of proregenerative factors contribute to liver regeneration through various cascades [2,36,56-59], as highlighted in section 2.2 and 2.3.

Simultaneously, the immune response is also fueled by pathogen-mediated inflammation emanating from the gut-liver axis [60,61]. PHx induces endotoxemia as a result of surgical ligation of part of the portal vein output, portal hypertension [25], and consequent perturbation of the intestinal mucosal barrier [62, 63]. This results in microbe-derived blood-borne lipopolysaccharide (LPS) – a pathogen-associated molecular pattern (PAMP) [ 64]- triggering liver regeneration by binding PRRs such as TLR4 on Kupffer cells [65]. LPS-PRR binding leads to Kupffer cell activation and the release of TNF-α and IL-6 [36,54,66]. The endotoxemia further facilitates the accumulation of platelets in the remnant liver [51,52], where the platelets locally orchestrate a pro-regenerative stimulus via degranulation and possibly sequestration by sinusoidal endothelial cells and hepatocytes [42] (section 2.3).

Thirdly, a shift in hepatocellular redox state is responsible for the onset of liver regeneration (Figure 1). The temporary pro-oxidative state post-PHx, which is characterized by an overproduction of reactive transients such as hydrogen peroxide (H2O2), can be caused by liver surgery encompassing ischemia/reperfusion [67,68], cholestasis [69] and other liver diseases [70], and a temporary post-PHx bile acid overload (section 3). Bai et al. showed that H2O2 at a specific intracellular concentration range promotes liver regeneration in rats during the first 24 hours after PHx [71], which has also been reported by others in mice [72,73], albeit with contradictory results [74].

Intracellular H2O2 acts as a cell cycle regulator that, depending on its intracellular concentration, signals quiescence or proliferation in hepatocytes. As illustrated in Figure 1, the process is regulated by the ERK-cyclin D1-pRB pathway [71] as well as Notch signaling [75]. ERK stands for extracellular regulated kinase (a MAPK), pRB signifies phosphorylated (i.e., activated) retinoblastoma protein (RB), and Notch is a cell surface protein that acts as an auxiliary mitogen in liver regeneration [76] (section 2.4). ERK signaling promotes cell cycle activity and ultimately proliferation, during which cyclin D1 and pRB, a cell cycle inhibitor in non-phosphorylated form [77,78], enable cell cycle progression and ultimately mitosis [79-81]. The transient increase in H2O2 levels is facilitated by temporary suppression of antioxidant enzymes and upregulation of pro-oxidant enzyme activity through nuclear factor erythroid 2-related factor 2 (NRF2) and other redox-active enzymes, particularly during the early regeneration phase [71]. In support of this, NRF2-null mice exhibit stalled liver regeneration [82]. To protect the hepatocytes from oxidative stress while undergoing reactive oxygen species (ROS)-mediated mitosis, heat shock proteins may be upregulated [83].

### 2.2 Cytokines in liver regeneration

Acting on the numerous environmental cues (section 2.1 and Figure 1), Kupffer cells become activated and release two important cytokines for liver regeneration, namely TNF-α and IL-6 [36,84,85]. While IL-6 is chiefly responsible for the mitogenic effects in hepatocytes, TNF-α predominantly serves an autocrine function in that it stimulates the production of IL-6 by Kupffer cells via nuclear factor kappa-light-chain-enhancer of activated B cells (NF-κB) [36,86] (Figure 2). However, it was shown that quiescent rat liver epithelial (LE6) cells also exhibit mitogenic activity following TNF-α stimulation, which proceeds through NF-κB and concurs with the upregulation of IL-6, signal transducer and activator of transcription 3 (STAT3, see below), and c-myc (an immediate-early gene that regulates cell cycle progression) [84,87,88], suggesting direct mitogenic signaling by TNF-α. Furthermore, the release of sphingosine 1-phosphate from activated platelets [89] prompts human sinusoidal endothelial cells to secrete IL-6 and thereby amplify the proliferative response in hepatocytes [90,91].

Hepatocytes normally reside in the quiescent (G0) phase, but after PHx enter the G1 phase following a multitude of stimuli, which includes IL-6 binding to its cognate receptor on the hepatocyte membrane [92,93]. As is illustrated in Figure 2, this triggers Janus kinase (JAK) signaling and the consequent transcription and translation of immediate-early target genes involved in DNA synthesis, cell proliferation, cellular hypertrophy, metabolic homeostasis, and cell survival by two major pathways [36,37,94,95]. Firstly, JAK activates the ERK-1/2 MAPK cascade via RAS and its complexation partners, culminating in cell proliferation [6]. Secondly, JAK activates STAT3 and the transcription of a plethora of immediate-early target genes [84,95,96], which mediate numerous liver regeneration-related processes [97].

### 2.3 Growth factors (complete mitogens) in liver regeneration

Liver regeneration is propagated by growth factors, whereby hepatocyte growth factor (HGF), vascular endothelial growth factor (VEGF), and ligands that bind epidermal growth factor (EGF) receptor (EGFR) occupy key roles [36,37, 98 -102]. Growth factor signaling encompasses several cell types and organs [36].

In the early phase of post-PHx liver regeneration, platelets accumulate in the remnant liver [103,104] via interactions with sinusoidal endothelial cells [42]. The platelets locally release an armament of mitogenic (HGF, EGF) and co-mitogenic growth factors (insulin-like growth factor-1 (IGF-1), VEGF, and platelet- derived growth factor (PDGF)) from their α-granules [47,105,106] as well as the proliferation-curtailing transforming growth factor β (TGF-β) [105,107]. Platelet degranulation also causes the release of non-growth factor mediators of liver regeneration [42], including serotonin [108] and nucleotides [105,106]. Moreover, extravasated platelets in the liver parenchyma induce proliferative AKT and ERK-1/2 signaling in hepatocytes through HGF, VEGF, and IGF [109,110].

An intricate signaling relationship exists between HGF and VEGF in the regenerating liver. HGF is released in its precursor form pro-HGF by stellate cells [111] and serotonin-activated sinusoidal endothelial cells [112]. Activated sinusoidal endothelial cells secrete VEGFA (hepatic VEGF) [112,113] that, upon autocrine binding to VEGF receptors (VEGFR1 [113] and VEGFR2 [114]), triggers the release of pro-HGF from sinusoidal endothelial cells [113]. At the same time, VEGFA drives the chemotaxis of bone marrow-derived sinusoidal progenitor cells to the liver, which are replete with HGF [ 115]. These progenitor cells not only differentiate into fenestrated sinusoidal endothelial cells as part of the regeneration process [99] but also locally release HGF [115]. Accordingly, rat plasma levels of (pro-)HGF increase rapidly by 10- to 20-fold following PHx [ 116]. Pro-HGF is converted to its active form by urokinase plasminogen activator (uPA) [117-119] that is hyperactivated after extracellular matrix (ECM) damage from the resection [2,34]. HGF acts in a paracrine and endocrine fashion with respect to hepatocytes, on which HGF binds its cognate receptor c-Met [120,122], inducing proliferative signaling and DNA synthesis through the ERK-1/2 MAPK pathway [123,124] and hepatoprotective signaling through AKT [125,126]. HGF-mediated proliferative signaling in rats was further shown to be amplified by the LPS [127] that is abundantly present in the enterohepatic circulation after PHx [60-64].

The HGF/c-Met pathway is amplified by ligands that bind epidermal growth factor receptor (EGFR), which include EGF, TGF-α, heparin-binding EGF-like growth factor (HBEGF), and amphiregulin [37,128,129], culminating in hepatocyte proliferation (Figure 2). Plasma levels of EGF, which is constitutively produced in the duodenum by the glands of Brunner [130], increase in response to elevated shear stress in the portal circulation [34] as well as norepinephrine signaling in the gut [131,132]. Plasma levels of norepinephrine increase within 20 min after PHx in rats [131,133] and may therefore fuel EGF signaling within the gut-liver signaling axis. TGF-α is produced by proliferating hepatocytes and relays proliferative signals to hepatocytes through an autocrine mechanism via EGFR [134-136]. HBEGF is produced by monocytes and macrophages [137,138] and converted to its active form by specific metalloproteinases [139]. In rats, plasma levels of this hepatocellular mitogen [140,141] are rapidly elevated after PHx [141,142] and expressed on or associated with sinusoidal endothelial cells and Kupffer cells as early as 90 minutes after PHx [141,143], reflecting early-onset proliferative cross-talk between the sinusoidal cells and EGFR-bearing hepatocytes via HBEGF. Amphiregulin is an autocrine growth factor and a mitogen for several cell types, including hepatocytes [144]. Accordingly, amphiregulin-null mice exhibit impaired hepatocellular proliferation [144]. The protein is induced in the early regeneration phase, triggered by prostaglandin E2 (PGE2) and IL-1β [144]. The latter was shown to be rapidly released after PHx in both mice and rats [145-147]. Similarly, PGE2 levels rise in the rat liver in the early phase of liver regeneration [148]. The release of IL-1β and PGE2 by Kupffer cells is stimulated by post-PHx endotoxemia [149]. Although both mediators induce amphiregulin and thus hepatocyte proliferation, IL-1β [145-147] and PGE2 concurrently inhibit liver regeneration. Whereas PGE2 inhibits liver regeneration through downmodulation of IL-6 by Kupffer cells [150], IL-1β inhibits expression of β-klotho and fibroblast growth factor receptor 4 (FGFR4), that together form the FGF15/19 receptor [151] (see section 3.3.1.3). Their inhibitory effects are evidently offset by the other proliferation - promoting processes after PHx [152].

Of the factors addressed above, HGF, EGF, TGF-α, HBEGF, and amphiregulin are classified as so-called ‘complete hepatic mitogens’ because these proteins trigger mitosis in cultured hepatocytes and induce liver hypertrophy and hepatocyte DNA synthesis in vivo [44]. By definition, ‘incomplete mitogens’ or ‘auxiliary mitogens’ are not mitogenic in cultured hepatocytes; they do not induce hepatocellular DNA synthesis and liver growth in vivo. Nonetheless, their inhibition (or inhibition of downstream targets) delays liver regeneration but does not abrogate it [44]. Some (potential) auxiliary mitogens are briefly addressed next.

### 2.4 Auxiliary mitogens in liver regeneration

The auxiliary mitogens TNF-α and IL-6 [36,84,85] (section 2.2), complement proteins [9] (section 2.1), serotonin [108] (section 2.3), and norepinephrine [153] (section 2.3) have been discussed above and elsewhere [2,36,37,44,56,98] in the framework of liver regeneration. Other putative auxiliary mitogens are the receptors of some of these compounds, which include VEGFR [113] (section 2.3), TNF receptor (TNFR) [93,154 ], serotonin receptors [108], and the norepinephrine receptor α1 adrenergic receptor [131]. Additional auxiliary mitogens that are thought to play a role in liver regeneration comprise FGF1 and FGF2 [155, 156], PDGF [157], macrophage inflammatory protein (MIP)-2 alpha (CXCL2) and its receptor IL-8 receptor beta (CXCR2) [158], the cell surface proteins NOTCH1 and JAG1 [76], leptin [159], insulin [160], hyaluronic acid [161-164], Wnt2 [114], and insulin-like growth-factor binding-protein 1 (IGFBP1) [165-167]. Their role has been reviewed in a broader context in [2,36,37,44,56,98]. The multiplicity of this non-exhaustive list of auxiliary mitogens clearly illustrates the complexity of signals that modulate post-PHx liver regeneration.

In addition to these putative auxiliary mitogens, some underexposed or more recently discovered auxiliary mitogens deserve to be highlighted. First, it was shown that PHx in mice leads to endoplasmic reticulum (ER) stress and the unfolded protein response (UPR) [168], which is triggered to resolve ER stress [169]. The UPR is mediated by inositol-requiring enzyme-1α (IRE1α) [170] and exacerbated by TNF-α and ROS [171]. This is also shown in Figure 2. As addressed in section 2.1 and 2.2, ROS and TNF-α are hallmarks of post-PHx liver regeneration and may therefore lie at the basis of the ER stress observed in hepatectomized mice [168]. More importantly, mice with *Ire1α*-null livers exhibit impaired hepatocyte proliferation and liver regeneration as a result of dampened IL-6-mediated STAT3 signaling [168]. It was further found that IRE1α interacts directly with STAT3 in the early phase of liver regeneration (6-12 hours), independently of IL-6 stimulation [168]. These results underscore the cytokine-driven as well as constitutive regulation of early, UPR-based mitogenic responses following PHx, and identify IRE1α as an auxiliary mitogen.

A related but relatively underexposed auxiliary mitogen is cyclooxygenase 2 (COX-2) [172], an enzyme that regulates prostaglandin synthesis. COX-2 is controlled by multiple processes [173] that occur during liver regeneration. These processes are also summarized in Figure 2. For instance, the post-PHx endotoxemia [60-63] drives COX-2 activation in rat Kupffer cells [174] that subsequently orchestrates cytokine signaling (section 2.2). Endotoxemia also positively affects hepatocyte COX-2 expression in mice [175]. Furthermore, ROS are a trigger for COX-2 upregulation [176,177]. COX-2-modulating pathways entail NF-κB [87] (section 2.2), ERK1/2 [36,178,179] (via e.g., transcriptional regulation of *PTGS2* [173], the gene encoding COX-2), and MEK in the MAPK pathway [6,178,179] (through e.g., the phospholipase A2 → arachidonic acid connection [173]), which are all established in post-PHx liver regeneration. As a result, COX-2 promotes hepatocyte proliferation during early regeneration, with maximum hepatocellular expression at 16 hours after PHx in rats [172]. The upregulation of COX-2 in regenerating livers is associated with decreased CCAAT-enhancer binding protein (C/EBP)α levels and increased expression of C/ EBPβ and C/EBPδ [172,180]. C/EBPs are transcription factors involved in cell proliferation, growth, differentiation, and metabolism [181,182]. C/EBPα inhibits proliferation [183,184] while the β and δ isoforms promote proliferation [179,185,186]. There is also a potential link between ER stress and COX-2. ER stress has been shown to stimulate murine COX-2 expression via activation of NF-κB and p38 MAPK [187]. In turn, p38 MAPK is induced by LPS and pro-inflammatory cytokines (e.g., TNF-α, IL-1β) [188-191] as well as by reactive transients such as hydrogen peroxide [191,192] and nitric oxide [193]. Similarly, NF-κB is under positive cytokine [194] and redox control [195]. These mechanisms connect post-PHx ER stress to COX-2 signaling in terms of hepatocyte proliferation, which are stimulated by liver regeneration-specific inflammation and redox-modulated processes.

An auxiliary role in liver regeneration has further been ascribed to microRNAs (miRNAs) [196,197], whereby some miRNA types are overexpressed (usually mildly; e.g., miR-21, miR-33, miR-153, and miR-743b [198,199]) while others are underexpressed (usually intensely; e.g., let-7b, let-7f, let-7g, miR-22a, miR-23b, miR-26a, miR-30b, and miR-122a [200,201]) after PHx [197]. Experiments in mice lacking the enzyme dicer 1 in the liver, which is responsible for generating miRNA [202], demonstrated that these animals exhibit a proliferative liver phenotype [203], indicating that miRNAs are essentially inhibitors of liver regeneration [200,204,205]. This inverse correlation is biochemically logical given that some miRNAs inhibit the translation of messenger RNA (mRNA) to a functional protein; in many cases cell cycle regulators [206,207] and mediators of proliferation [200,208,209]. However, there are several examples of miRNAs that are upregulated during liver regeneration yet amplify the regenerative response, suggesting that these transcriptomic regulators inhibit repressors of hepatocellular proliferation. Specific examples include miR-21 [207,210-213], miR-221 [214], miR-378 [210], and miR-382 [215].

The expression patterns of miRNA are species-dependent [216], dynamic over time, and the peak expression levels do not temporally overlap [198,217,218], underscoring the phasic nature and pleiotropic signaling of miRNAs as has been reported for cytokines (section 2.2) and growth factors (section 2.3). In that respect, the temporal heterogeneity of intrahepatic and plasma cytokine levels [36,37,56,84] are partly responsible for the differential miRNA expression profiles inasmuch as cytokines modulate miRNA expression [219]. In addition to changes in their quantitative expression, the miRNAs, which are associated with polysomes (mRNA-ribosome complexes formed during active translation), also exhibit spatial translocation during liver regeneration [197]. Corroboratively, the expression levels of the miRNAs let-7a, miR-21, miR-195, and miR-215 increased in the membrane-bound polysomes relative to the free polysomes after PHx [197].

## 3. Bile acids co-regulate post-hepatectomy liver regeneration in the early phase

Studies in the recent years have shown that bile acid metabolism and signaling are directly involved in the liver regeneration process [21,28,220,222]. After PHx, the remaining liver is subject to acute overload of bile acids returning via the portal circulation [22,24-26]. Bile acids are complete mitogens by virtue of their binding to nuclear receptors or activating intracellular signaling pathways [19,20]. During the regeneration phase, the liver also activates multiple adaptive mechanisms to prevent bile acid toxicity and restore bile acid homeostasis since prolonged exposure to certain bile acids at higher concentrations may promote liver injury or tumorigenesis in chronic liver injury-repair processes [223]. The effect of bile acid metabolism on liver regeneration, regulation of liver proliferation by bile acid signaling, and the mechanisms regulating bile acid homeostasis during regeneration are further discussed in the following sections.

### 3.1 Bile acid synthesis and cycling through the enterohepatic circulation

#### 3.1.1 Bile acid synthesis

**Figure 3.**
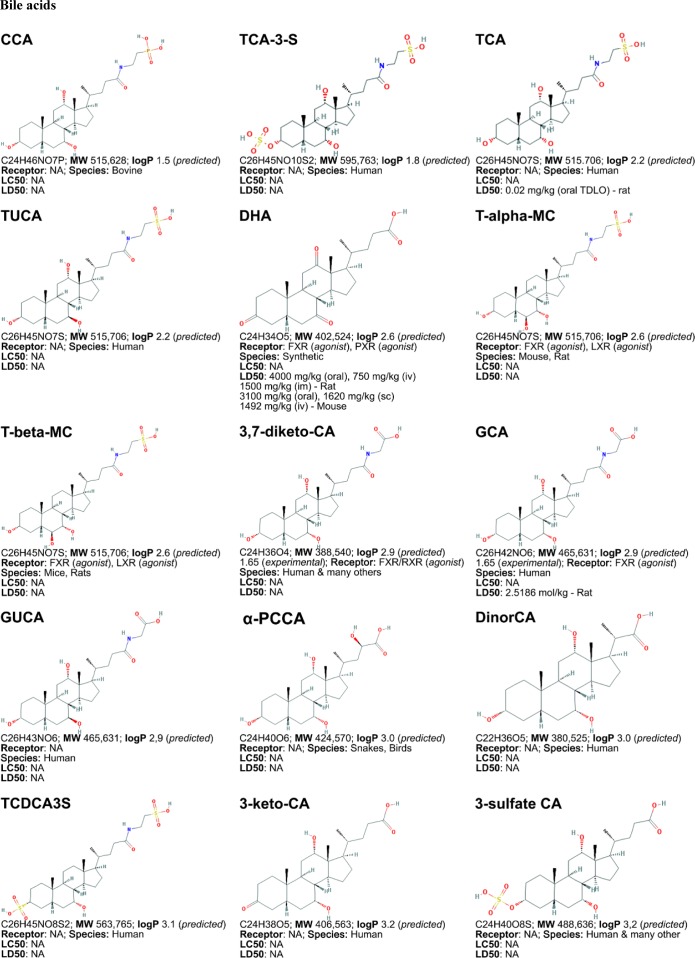

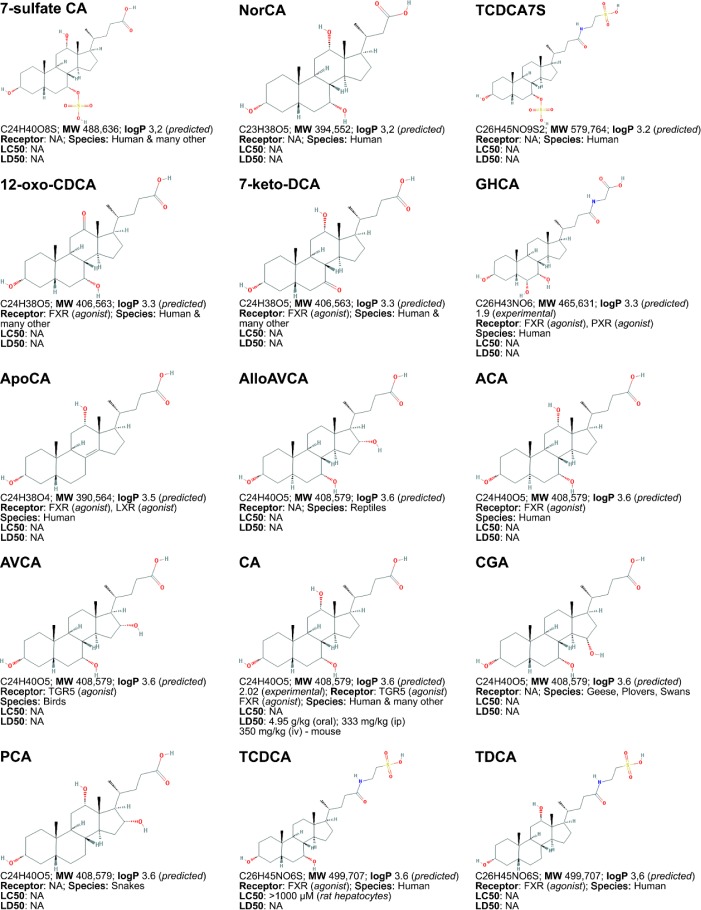

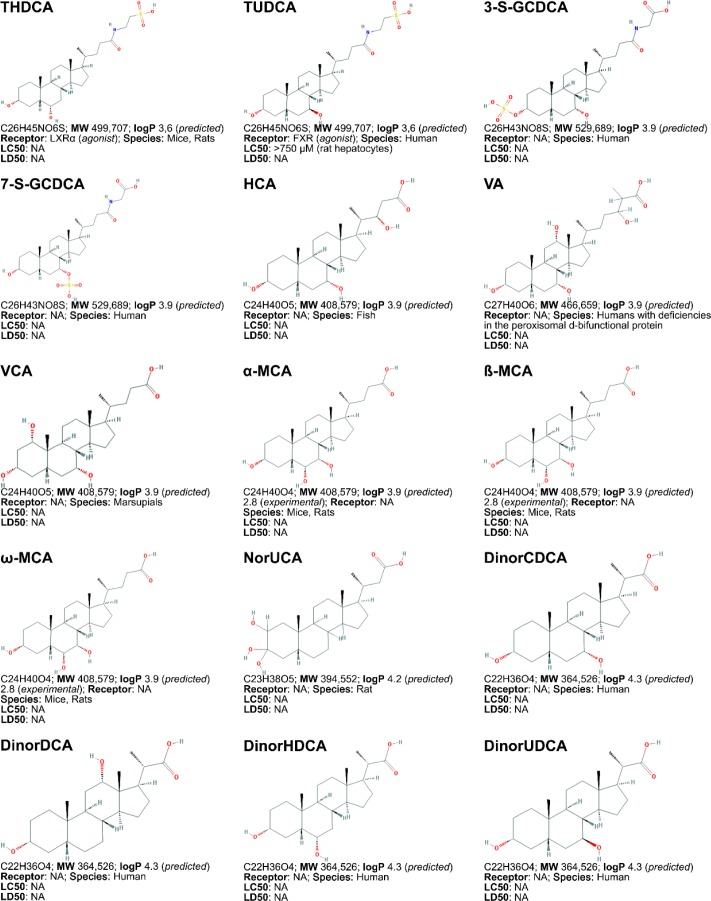

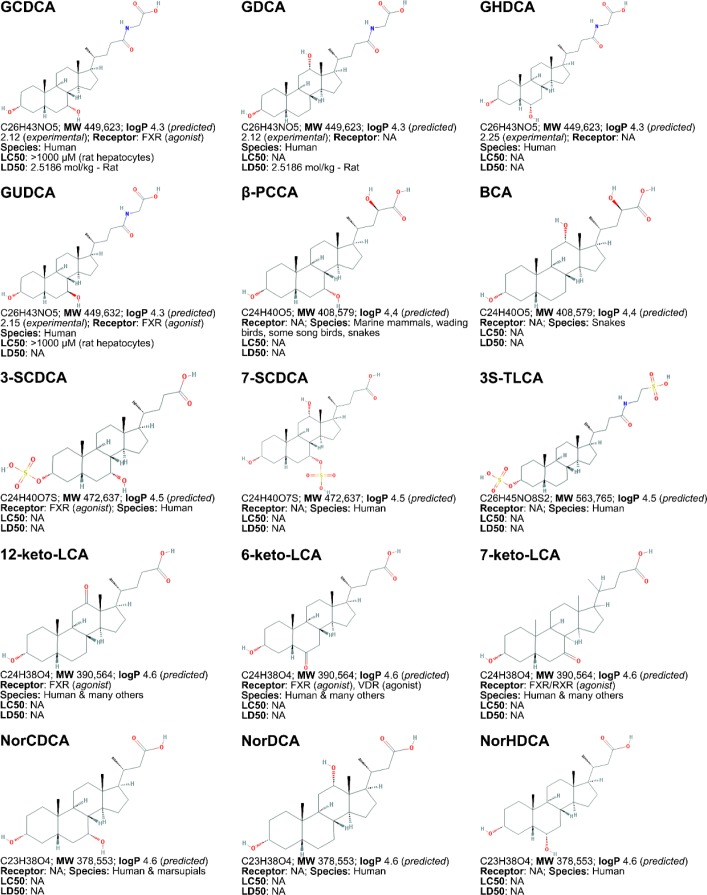

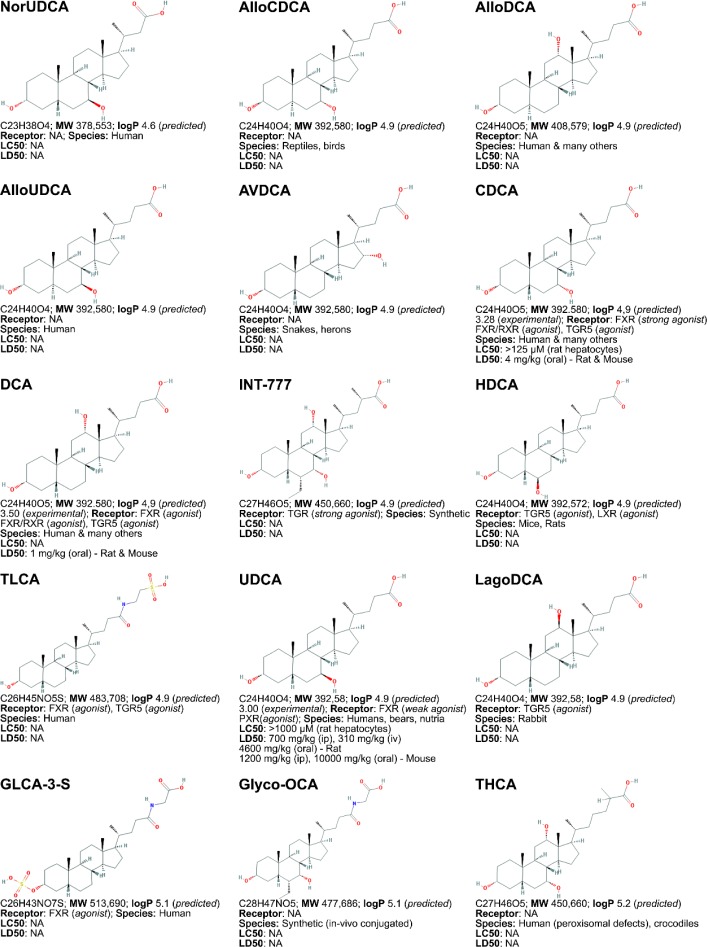

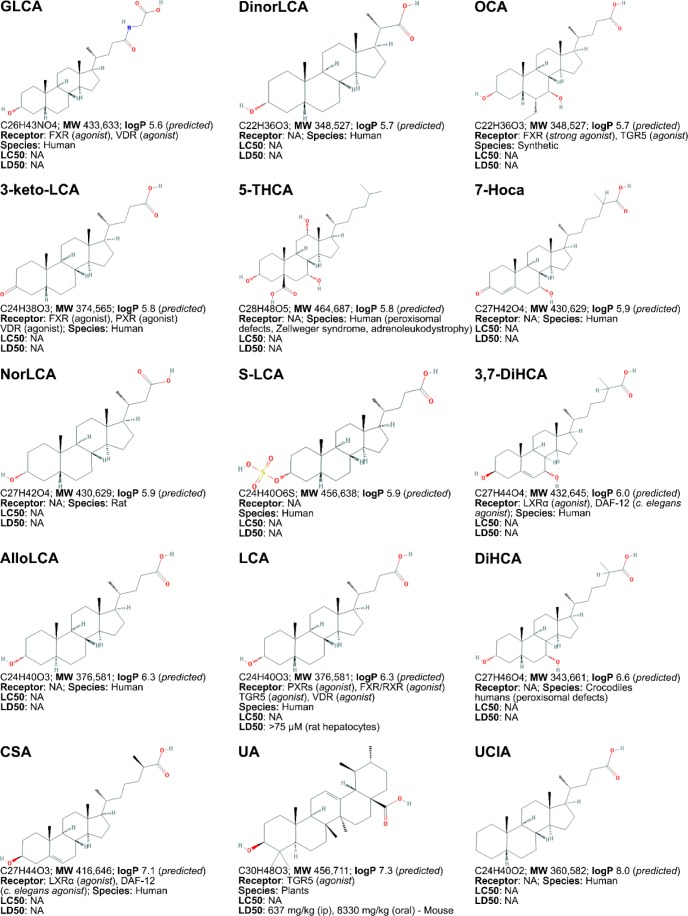

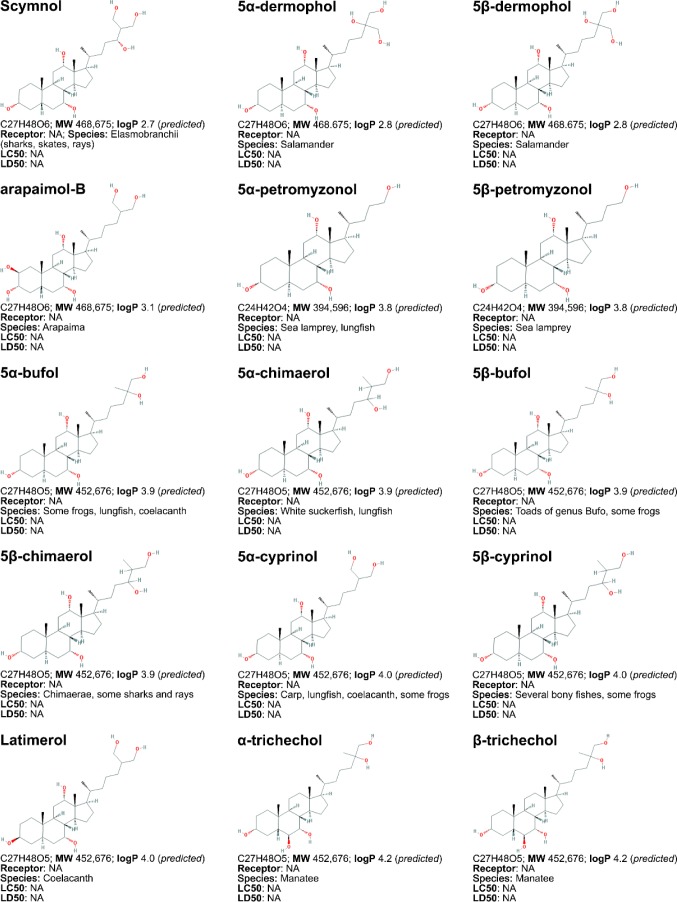

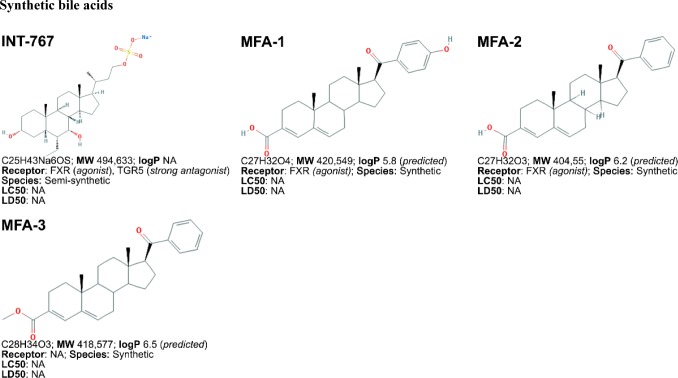
**Non-exhaustive list of bile acid species and bile acid analogues, chemical properties, and toxicity**. LogP (octanol:water partition coefficient) values were retrieved from PubChem and were predicted with XLogP2 or XLogP3 software. The 50% lethal concentration (LC50, used for in vitro data) and 50% lethal dose (LD50, used for in vivo data) were obtained from the material safety data sheets (retrieved from the Cayman Chemicals and Spectrum Chemical website) and the Toxicological Data Network (TOXNET, https://toxnet.nlm.nih.gov/) as well as available literature [544] [544]. Abbreviations (bile acids excluded): NA: information not available; iv: intravenous; ip: intraperitoneal; MW: molecular weight; sc: subcutaneous; TDLO; the lowest dose causing a toxic effect. Abbreviations (bile acids): 12-keto-LCA: 12-ketolithocholic acid / 12-oxolithocholic acid; 12-oxo-CDCA: 12-oxochenodeoxycholate / 12-oxochenodeoxycholic acid; 3,7-DiHCA : 3,7-dihydroxy-5-cholestenoic acid; 3,7-diketo-CA : 3,7-diketocholanic acid / 3,7-dioxhocholanoic acid; 3-keto-CA: 3-ketocholic acid / 3-oxocholic acid; 3-keto-LCA: 3-ketolithocholic acid / dehydrolithocholic acid; 3-SCDCA: chenodeoxycholic acid 3-sulfate; 3-S-GCDCA: glycochenodeoxycholic acid 3-sulfate; 3S-TLCA: taurolitocholate sulfate / taurolithocholic acid 3-sulfate; 3-sulfate CA: cholic acid 3-sulfate; 5-THCA: trihydrocoprostanic acid / (3alpha,5beta,7alpha,12alpha)-3,7,12-trihydroxycholestane-5-carboxylic acid; 6-keto-LCA: 6- ketolithocholicacid;7-Hoca: 7α-hydroxy-3-oxo-4-cholestenoicacid;7-keto-DCA:7-ketodeoxycholicacid;7-keto-LCA:7- ketolithocholic acid / nutriacholic acid; 7-SCDCA: chenodeoxycholic acid 7-sulfate; 7-S-GCDCA: glycochenodeoxycholic acid 7- sulfate; 7-sulfate CA: cholic acid 7-sulfate; α-MCA: α-muricholate / α-muricholic acid / α-hyocholic acid; α-PCCA: α-phocaecholate / alpha-phocaecholic acid; β-MCA: β-muricholate / β-muricholic acid / β-hyocholic acid; β-PCCA: β-phocaecholate / phocaecholic acid; ω-MCA: ω-muricholate / ω-muricholic acid / ω-hyocholic acid; ACA: allocholate / allocholic acid; AlloAVCA: alloavicholate / alloavicholic acid; AlloCDCA: allochenodeoxycholate / allochenodeoxycholic acid; AlloDCA: allodeoxycholate / allodeoxycholic acid; AlloLCA: allolithocholic acid; AlloUDCA: alloursodeoxycholic acid; ApoCA: apocholate / apocholic acid; AVCA: avicholate / avicholic acid; AVDCA: avideoxycholate / avideoxycholic acid; BCA: bitocholate / bitocholic acid; CA: cholate / cholic acid; CCA: ciliatocholate / ciliatocholic acid; CDCA: chenodeoxycholate / chenodeoxycholic acid; CGA: cygnocholate / cygnocholic acid; CSA: cholestenoic acid; DCA: deoxycholate / deoxycholic acid; DHA: dehydrocholate / dehydrocholic acid; DiHCA: dihydroxycoprostanoic acid; DinorCA: dinorcholic acid; DinorCDCA: dinorchenodeoxycholic acid; DinorDCA: dinordeoxycholic acid; DinorHDCA: dinorhyodeoxycholic acid; DinorLCA: dinorlithocholic acid; DinorUDCA: dinorursodeoxycholic acid; GCA: glycocholate / glycocholic acid; GCDCA: glycochenodeoxycholate / glycochenodeoxycholic acid; GDCA: glycodeoxycholate / glycodeoxycholic acid; GHCA: glycohyocholate / glycohyocholic acid; GHDCA: glycohyodeoxycholate / glycohyodeoxycholic acid; GLCA: glycolithocholate / glycolithocholic acid; GLCA-3-S: glycolithocholate 3-sulfate / glycolithocholic acid 3-sulfate; Glyco-OCA: glyco-obeticholic acid; GUCA: glycoursocholate / glycoursocholic acid; GUDCA: glycoursodeoxycholate / glycoursodeoxycholic acid; HCA: hemulcholate / hemulcholic acid; HDCA: hyodeoxycholic acid / murideoxycholic acid; LagoDCA: lagodeoxycholic acid; LCA: lithocholate / lithocholic acid; NorCA: norcholate / norcholic acid; NorCDCA: norchenodeoxycholic acid; NorDCA: nordeoxycholic acid; NorHDCA: norhyodeoxycholic acid; NorLCA: norlithocholic acid; NorUCA: noruroscholic acid; NorUDCA: norursodeoxycholic acid; OCA: obeticholic acid / ocaliva / 6-ethylchenodeoxycholic acid / INT-747; PCA: pythocholic acid; S-LCA: lithocholic acid 3-sulfate; T-alpha-MC: tauro-α-muricholic acid; T-beta-MC: tauro-β-muricholic acid; TCA: taurocholate / taurocholic acid; TCA-3-S: taurocholate 3-sulfate / taurocholic acid 3-sulfate; TCDCA: taurochenodeoxycholate / taurochenodeoxycholic acid; TCDCA3S: taurochenodeoxycholic acid 3-sulfate; TCDCA7S: taurochenodeoxycholic acid 7-sulfate; TDC(A): taurodeoxycholate / taurodeoxycholic acid; THCA: trihydrocoprostanic acid / coprocholic acid / 3,7,12-trihydroxycholestan-26-oic acid; THDCA: taurohyodeoxycholate / taurohyodeoxycholic acid; TLCA: taurolithocholate / taurolithocholic acid; TUCA: tauroursocholate / tauroursocholic acid; TUDCA: tauroursodeoxycholate / tauroursodeoxycholic acid; UA: ursolic acid; UCA: ursocholic acid; UClA: ursocholanic acid; UDCA: ursodeoxycholate / ursodeoxycholic acid; VA: varanic acid; VCA: vulpecholate / vulpecholic acid.

Daily, 0.2 to 0.6 g of bile acids (Figure 3, placed at the end of the manuscript) is synthesized from cholesterol in the human liver via two pathways: the neutral pathway and the acidic pathway [224]. Bile acid synthesis pathways involve multi-step reactions catalyzed by enzymes in the endoplasmatic reticulum, mitochondria, cytoplasm, and peroxisomes. The enzyme cholesterol 7α-hydroxylase (CYP7A1) catalyzes the first rate-limiting step in the neutral pathway and converts cholesterol into 7α-hydroxycholesterol, which eventually leads to the synthesis of the primary bile acids cholic acid (CA) or chenodeoxycholic acid (CDCA) [225,226]. In the acidic pathway, the enzyme 27α-hydroxylase (CYP27A1) converts cholesterol to 27α-hydroxycholesterol, which leads to the synthesis of CDCA [224]. After excretion into the biliary system, bile acids are first deconjugated and then biotransformed by enteral bacteria through phase I reactions (oxidation, hydroxylation), forming secondary bile acids, including deoxycholic acid (DCA), lithocholic acid (LCA), and ursodeoxycholic acid (UDCA) [227-229]. Ninety five percent of all biliary excreted bile acids are resorbed and transported back to the liver via the portal vein, also referred to as the enterohepatic circulation [230]. However, most LCA is not recycled and the small amount of circulating LCA is rapidly conjugated by sulfation, a phase II reaction, and secreted into the biliary system [228]. Sulfation increases bile acid solubility, as a result of which sulfated LCA is less likely to be intestinally reabsorbed [231]. For this reason, the human bile acid pool mainly consists of the primary bile acids CA and CDCA and the secondary bile acid DCA. The liver efficiently conjugates both primary and secondary bile acids to taurine or glycine (ratio 3:1 in humans) by amidation, then commonly referred to as bile salts, which promotes solubility and prevents passive diffusion across cell membranes [232]. Throughout this review, the term ‘bile acids’ is used for both bile acids and bile salts.

In the mouse and rat liver, the majority of CDCA is further converted to the more hydrophilic muricholic acids (MCAs) α-muricholic acid and β-muricholic acid. Consequently, the mouse and rat bile acid pool consists of approximately equal amounts of CA and MCAs, with relatively low levels of CDCA. While in humans glycine-conjugated bile acids are most common, most bile acids in mice and rats are conjugated with taurine [233]. Thus, the mouse bile acid pool is much more hydrophilic compared to the human bile acid pool. This is the main reason for the direct cytotoxicity of bile acid during cholestasis in humans but not mice [234,235]. Nevertheless, similar mechanisms of bile acid metabolism likely apply to most species.

#### 3.1.2 Bile acid transport in the enterohepatic circulation

Bile acids produced in hepatocytes are efficiently secreted into the bile and stored in the gallbladder. Upon food intake, the gallbladder contracts in response to cholecystokinin secreted by the epithelial cells in the duodenum, causing bile acids to be released into the small intestine [236]. In the small intestine, bile acids emulsify dietary lipids to form micelles, allowing pancreatic lipases to hydrolyze lipids for absorption.

**Figure 4.**
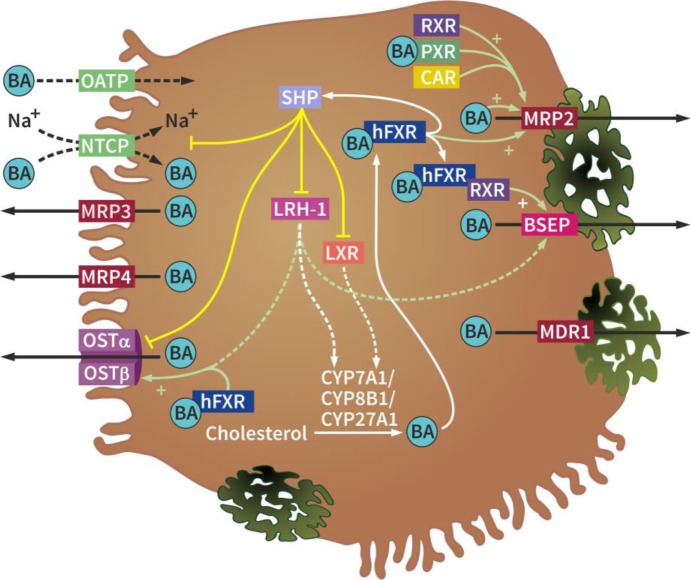
**Hepatocellular bile acid transporters**. Basolateral import of bile acids is mediated by NTCP (Na^+^-dependent) and OATP isoforms (Na+-independent). Bile acids are exported through the basolateral exporters MRP3, MRP4, and the OSTα and OSTβ heterodimer and through the canalicular exporters BSEP, MRP2, and possibly MDR1. Bile acids regulate their own efflux through hepatic farnesoid X receptor (hFXR). Bile acid-activated hFXR induces the OSTα and OSTβ heterodimer and MRP2 and, as heterodimer with RXR, BSEP. hFXR also activates SHP that inhibits the importers NTCP, the OSTα and OSTβ heterodimer, LXR, and LRH-1. LXR and LRH-1 normally inhibit CYP7A1, CYP8B1, and CYP27A1, but because of SHP activation by hFXR and consequent inhibition of LRH-1 and LXR, bile acids are synthesized from cholesterol. Additionally, LRH-1 normally stimulates the expression of BSEP and the OSTα and OSTβ heterodimer, while induction of SHP by hFXR results in inhibition of those exporters. Abbreviations: BA, bile acid; BSEP, bile salt export pump; CAR, constitutively active/androstane receptor; CYP, cytochrome p450; hFXR, hepatic farnesoid X receptor; LRH-1, liver receptor homolog 1; LXR, liver X receptor; MDR1, multidrug resistance associated protein 1; MRP3/4, multidrug resistance protein 3 and 4; NTCP, Na+-taurocholate co-transporting polypeptide; OATP, organic anion transporting polypeptide; OSTα/β, organic solute transporter alpha/beta; PXR, pregnane X receptor; RXR, retinoid X receptor; SHP, small heterodimer partner.

After reabsorption by enterocytes, bile acids in the portal circulation are imported into hepatocytes across the basolateral membrane, after which they are secreted into the bile canaliculi, a process referred to as first pass metabolism (summarized in Figure 4). The liver first pass extraction rate for conjugated bile acids is about 90%, with little bile acids spilled into the systemic circulation. The Na^+^-dependent taurocholate transporter (NTCP) is a major bile acid uptake transporter in the basolateral membrane of hepatocytes [237-241]. In addition, organic anion transporter (OATP) isoforms mediate Na^+^-independent bile acid uptake at the basolateral membrane of the hepatocytes. At the canalicular side of the hepatocytes, the bile salt export pump (BSEP, *ABCB11 /* *Abcb11*; human / rodent gene) mediates bile acid secretion against a concentration gradient. Consequently, canalicular bile acid export is a rate-limiting step in bile formation [242]. The multidrug resistance-associated protein-2 (MRP2, *ABCC2 / Abcc2*) can also mediate the canalicular secretion of certain sulfated and conjugated bile acids, besides bilirubin conjugates, glutathione, and drugs [243]. Hepatobiliary free cholesterol secretion into the bile is mediated by the ATP-binding cassette transporters ABCG5 and ABCG8 [244]. Phosphatidylcholine, the major phospholipid in the bile, is secreted via the multi-drug resistance protein (MDR3, *ABCB4 / Abcb4*) [245]. Cholesterol, bile acids, and phospholipids are the major constituents of bile. They form micelles in the canaliculi to increase cholesterol solubility and decrease bile acid damage to the bile duct.

In the intestine, bile acids are imported into enterocytes via the apical sodium-dependent bile acid transporter (ASBT, SCLC10A2 / Sclc10a2) [246] and subsequently excreted into the portal circulation via the organic solute and steroid transporter (OST)α and OSTβ heterodimer [247,248]. Whereas most of the conjugated bile acids are efficiently reabsorbed in the small intestine via active transport systems, some unconjugated primary bile acids and secondary bile acids, mainly DCA and to a much less extent LCA, can also be reabsorbed in the colon via passive diffusion and returned to the liver via the portal circulation.

#### 3.1.3 Bile acid-activated signaling

Besides the digestive function, bile acids are also signaling molecules that regulate various physiological and pathophysiological processes, including metabolic homeostasis, tumorigenesis, and immunity. In the enterohepatic system, bile acids exert regulatory functions by activating either intracellular ligand-activated nuclear receptors or cell surface receptors that activate intracellular signaling [249-251]. One of the major functions of the nuclear receptors is to maintain bile acid homeostasis through coordinated regulation of bile acid synthesis, transport, and detoxification.

**Figure 5.**
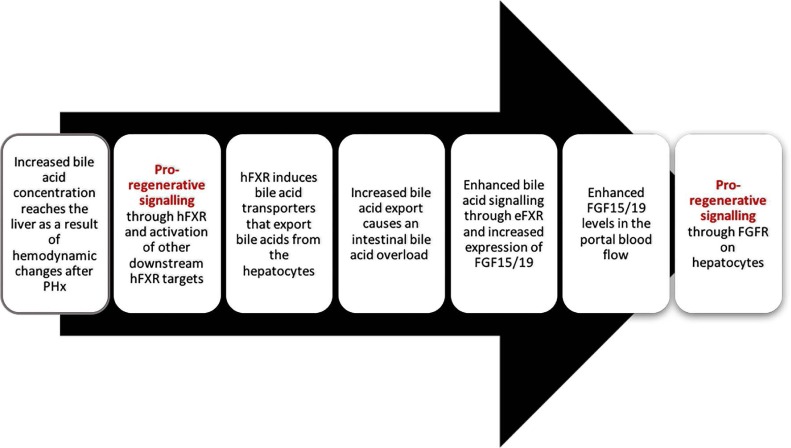
Chronological flowchart of mitogenic signaling by bile acids.

**Figure 6:**
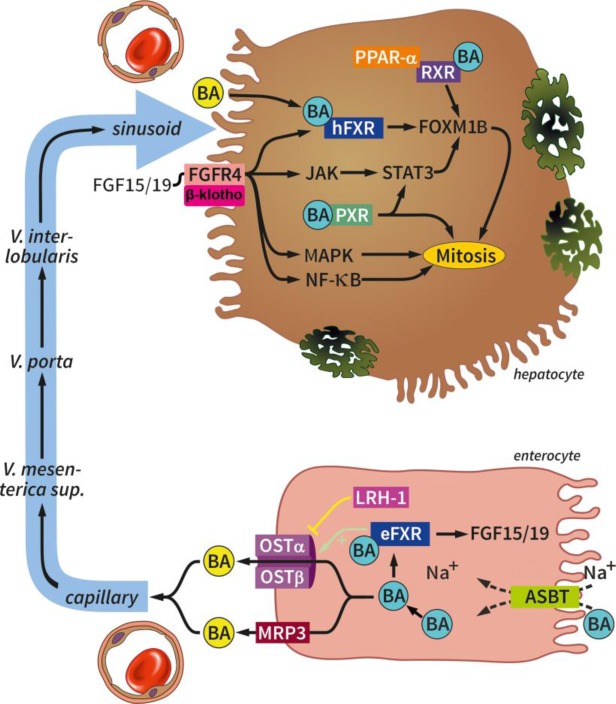
Hepatocyte-enterocyte interplay after PHx. Bile acids are taken up in the intestine by the enteral importer ASBT and exported into the portal circulation by the OSTα and OSTβ heterodimer and MRP3. In the enterocyte, bile acids activate eFXR that activates the OSTα and OSTβ heterodimer and induces the transcription of FGF15/19. FGF15/19 binds to the FGFR4/β-klotho receptor complex that in turn stimulates mitosis through pathways involving hFXR/FOXM1B, JAK/STAT3/FOXM1B, MAPK, and NF-κB. In the hepatocyte, bile acids can bind PXR and hFXR that stimulate mitosis through STAT3 and FOXM1B, respectively. Bile acids bound to RXR complexed with PPAR-α can also induce hepatocyte proliferation. Abbreviations: BA, bile acid; ASBT, apical sodium dependent bile acid transporter; FGF15/19, fibroblast growth factor 15/19; eFXR, enteral farnesoid X receptor; LRH-1, liver receptor homolog 1; OSTα/β, organic solute transporter alpha/beta; MRP3, multidrug resistance protein 3; FGFR4, fibroblast growth factor receptor 4; JAK, Janus kinase; ERK, extracellular signal-regulated kinase; MAPK, mitogen-activated protein kinase; STAT3, signal transducer and activator of transcription; NF-κB, nuclear factor kappa-light-chain-enhancer of activated B-cells; FOXM1B, forkhead box M1B; hFXR, hepatic farnesoid X receptor; PXR, pregnane X receptor; RXR, retinoid X receptor; PPAR-α, peroxisome proliferator-activated receptor alpha.

The best studied nuclear receptor, farnesoid X receptor (FXR), is primarily expressed in the liver and intestines and, as shown in Figure 5 and Figure 6, distinct pathways are initiated by hepatocellular FXR (hFXR) [252] and enterocytic FXR (eFXR) [253] that are involved in post-PHx liver regeneration and bile acid homeostasis [28,254]. In the liver, hFXR regulates bile acid metabolism through a feedback and feedfoward mechanism. Once activated by bile acids, hFXR mediates a negative feedback loop through inhibition of the bile acid synthesis genes *CYP7A1, CYP8B1, CYP27A1,* and the bile acid uptake transporter NTCP [224,255] (Table 1). Additionally, bile acid-activated hFXR regulates bile acid homeostasis through a feedfoward mechanism by stimulating the expression of the bile acid efflux transporter [256]. In the intestine, eFXR inhibits ASBT and induces OSTα and OSTβ to reduce bile acid accumulation in enterocytes. Bile acid activation of eFXR also induces the endocrine hormone fibroblast growth factor 15 (FGF15, FGF19 in humans) (Table 1). FGF15 can bind to its cognate receptor FGFR4 on the surface of hepatocytes and inhibits CYP7A1 and bile acid synthesis through various mechanisms activated by FGFR4, such as the MAPK-ERK pathway.

Recent studies have shown that this bile acid activated gut-to-liver signaling axis plays an important part in the liver regeneration process [22,257] (Figure 5). In response to bile acid overload, pregnane X receptor (PXR, section 3.3.3) and constitutively active/andostane receptor (CAR, section 3.3.4) play important roles in activating numerous bile acid detoxification mechanisms. More recently, these two nuclear receptors have also been implicated in the regulation of liver regeneration.

**Table 1.**
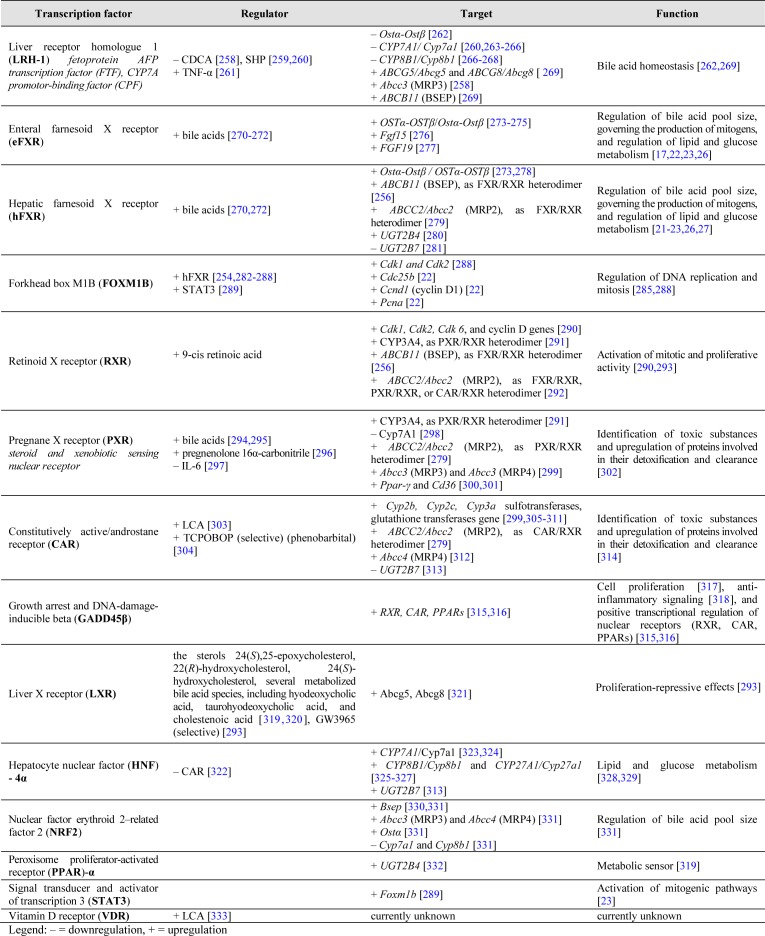


### 3.2 Impact of bile acid pool alteration on liver regeneration

PHx causes acute but temporary bile acid overload in the liver [22,24-26] and the systemic circulation [21]. The bile acid overload in rat hepatocytes occurs within several hours after PHx [26] and peaks around 24 hours [22]. Bile acids are cytotoxic at high concentrations [334,335], and hepatic bile acid levels are quite quickly restored to pre-PHx status, namely within 24-48 hours after resection [22,24,26]. Initial evidence suggesting that bile acid signaling was involved in liver regeneration came from studies showing that experimentally altering the bile acid pool size could significantly modulate the liver regeneration rate in mice after PHx [28]. Rats that lack intestinal bile acid reabsorption due to external bile drainage showed lower proliferative activity in the liver and slower liver regrowth after PHx compared to rats that underwent bile drainage into the duodenum before PHx [21,221]. Huang et al. [28] further showed that mice that had been supplemented with 0.2% CA for 5 days exhibited more rapid liver regeneration, while mice fed the bile acid sequestrant cholestyraminefor5daysshoweddelayedliverregeneration after PH [28]. In rats that had undergone PHx, dietary supplementation with the bile acids UDCA and tauroursodeoxycholate induced hepatocyte proliferation [220]. A more recent clinical study showed that patients who underwent hemihepatectomy without external bile drainage had about ~3-fold more liver regrowth volume on day 7 than patients who underwent hemihepatectomy with external bile drainage [222,336].

The importance of bile acids in liver regeneration is also manifested in more circumstantial evidence. For example, liver regrowth elicited by CA feeding was reduced in mice lacking the basolateral bile acid exporter multidrug resistance-associated protein 3 (MRP3, *Abcc3*, Figure 6) [337, 338], and this was associated with decreased portal bile acid concentration and FXR activation [ 339]. Similarly, delayed regeneration was also reported in mice lacking CYP27A1 [340] and in mice lacking ASBT [21,339, 341 - 343]. Moreover, transcriptomic analysis revealed that activator protein 1 (AP-1), a heterodimeric early response transcription factor composed of c-Jun and c-Fos and in control of cell cycle activity and proliferation [344,345], is downregulated in the absence of bile acids in hepatectomized rats [21]. Finally, experiments with ‘hypertransgenic’ FRGN mice bearing humanized livers demonstrated a positive correlation between the size of the intrahepatic bile acid pool and the degree of liver growth [29]. These studies altogether support the prominent role of bile acids in post-PHx liver regeneration.

### 3.3 The role of nuclear receptors in post-hepatectomy liver regeneration and bile acid signaling

Nuclear receptors are transcription factors that serve as metabolic sensors in that they bind xenobiotics and metabolic intermediates (e.g., fatty acids, sterols, bile acids [319]) to regulate their levels and signaling effects as well as a plethoraofmetabolicpathways.Afractionofthemitogenic signalsrelayedbybileacidsaremoderatedthroughnuclear receptors [19,20] - a process that is associated with translocation of specific bile acids from the cytoplasm to the nucleus [24]. The nuclear receptors involved in liver regeneration can exert bidirectional effects on hepatocellular proliferation, entailing both promotion and suppression of liver regeneration [19,20]. In terms of PHx, the promotors encompass FXR [28,254, 346], retinoid X receptor (RXR) [347, 348], PXR [ 349], and CAR [28,350 ]andtheircommonheterodimerpartner,retinoidX receptor (RXR) [347,348]. Contrastingly, the suppressors encompass the peroxisome proliferator-activated receptors (PPARs) [ 351] PPAR-α [352] and PPAR-γ [353,354]. Genetic ablation of liver X receptor (LXR) in mice has no notable effect ofpost-PHxliverregeneration.However,thereceptorisnot necessarily mitogenically neutral since treatment with its agonist GW3965 in wild type mice hampers several effectors of proliferation and moderately retards liver regrowth [293]. LXR hence exhibits an inclination to repress liver regeneration. Liver receptor homologue 1 (LRH-1), alternatively referred to as fetoprotein AFP transcription factor (FTF) and CYP7A promoter-binding factor (CPF), is also a nuclear receptor in the liver [258]. The activities of LRH-1 revolve mainly around bile acid homeostasis [262,269] rather than induction of proliferation during post-PHx liver regeneration [355]. LRH-1 therefore fulfills a detoxification function during bile acid overload. A similarroleiscurrentlyascribedtothevitaminDreceptor (VDR). This nuclear receptor is also expressed in hepatocytes [356]andregulatesbileacidmetabolismthroughCYP-based [333,357] and sulfotransferase-based bile acid detoxification [358-360] and bile acid transporter control [361].

FXR, LXR, PXR, PPARs, and LRH-1 are metabolic sensors in that they can be bound and activated by bile acids [319], and consequently regulate the levels and signaling intensity of the end-products through downstream effectors. Although CAR and VDR do not bind bile acids with the exception of LCA (in vivo) [303,333], the receptors are amenable to bile acid signaling since they are activated by ancillary bile acid-induced processes to mediate biological effects that are, or could be, pertinent in liver regeneration [319,333,357-363].

#### 3.3.1 Farnesoid X receptor signaling

Of all nuclear receptors, FXR is the best studied in the context of post-PHx liver regeneration. The bile acids CDCA, DCA, and LCA are physiological ligands of FXR [270-272], with a relative FXR potency of CDCA > DCA = LCA > CA [271]. The prime function of FXR is to regulate bile acid metabolism and homeostasis [319,364-366]. On top of that, FXR governs lipid and glucose metabolism [319,364-366], and which are essential in liver regeneration [367,368]. With respect to bile acid homeostasis, the receptor regulates genes that control bile acid synthesis, secretion, uptake, transport, and endobiotic metabolism [369-372]. FXR is expressed at high levels in the liver [252] and intestines [253] and as such constitutes an integral part of enterohepatic communication, especially in case of liver regeneration. The individual effects of liver hFXR and intestinal eFXR will be described in detail in the following sections.

##### 3.3.1.1 Mitogenic and metabolic signaling triggered by bile acids in the liver through hepatocellular farnesoid X receptor

Liver-specific FXR-null mice exhibit stalled liver regeneration compared to animals with functional hepatic FXR [28,254]. Similarly, FXR activity is significantly impaired and its downstream target genes *Shp*, *Cyp7a1* (section 4.2), and *Abcb11* (BSEP) (section 4.3.3) are afflicted in transgenic mice that overexpress the endogenous FXR inhibitor sirtuin 1 (SIRT1) [373]. PHx in these mice resulted in a debilitated regenerative response and bile acid-induced toxicity [373].

Mechanistically, activated hFXR signals proliferation through FOXM1B [254,282-288] (Table 1), a transcription factor that regulates DNA replication and mitosis by stimulating the expression of proteins that are responsible for cyclin-dependent kinase 2 (CDK2) and CDK1 cell cycle activity [285,288]. CDK2 is crucial for the G1/S transition [374], whereas CDK1 enables cell cycle progression from the S-phase to mitosis (M-phase) [375]. CDK2 complexes with cyclins and other CDKs to activate RB [376,377] (section 2.1) and is under positive control of CDC25a [376], which in turn is antagonized by the cyclin-dependent kinase inhibitor p21 378[]. Hepatectomized *Foxm1b*^−/−^ mice showed increased hepatocellular nuclear p21 levels, reduced Cdc25a expression, and consequently decreased activation of CDK2 and RB [285]. Moreover, *Foxm1b*^−/−^ mice exhibited increased levels of phosphorylated CDK1 [285], which is normally dephosphorylated by CDC5B to promote M-phase progression [379-381], as well as no expression of nuclear CDC25B protein [285]. These findings demonstrate that hepatocyte proliferation is controlled at the level of the cell cycle by hepatocellular FOXM1B, which is induced by hFXR that in turn is activated by bile acids (Table 1).

In addition to mitogenic signaling, hFXR-steered liver regeneration after PHx may be metabolically supported as a result of hFXR activation of the pyruvate dehydrogenase lipoamide kinase isozyme 4 (PDK4) [27,382] that resides in mitochondria and regulates pyruvate metabolism as part of gluconeogenesis [ 383]. PDK4 is induced by hyponutrition and facilitates the utilization of alternative carbon sources for gluconeogenesis [384]. PDK4 was upregulated following FXR activation and downregulated after FXR knockdown in HepG2 cells [27]. Induction of such a switch is analogous to the Warburg effect in highly proliferative cancer cells, typified by a metabolic shift towards converting glucose and certain amino acids into biomass [385,386]. Activation of FXR in human hepatocellular carcinoma (HepG2) cells by the agonists GW4064 and CDCA resulted in increased PDK4-mediated of lactic acid, pyruvic acid, and glucose-6-phostate (i.e., citric acid cycle metabolites), increased glucose uptake and glycolysis, and augmented production of glycine and serine [27], which are important in cell proliferation [387,388]. Conversely, siRNA knock-down of FXR abrogated or inverted these processes [27]. Cells in which FXR was knocked down proliferated poorly and did not exhibit the PDK4-mediated metabolic reprogramming [27]. It should be noted that, although FXR-induction of PDK4 is in line with anabolic demand during the liver regrowth phase, hitherto no study has shown that PDK4 enzyme activity is increased after PHx in wild type and FXR-null mice [27]. These studies are still needed to establish a role of the FXR-PDK4 axis in liver regeneration, especially given that PDK4 is transcriptionally regulated by complex signaling mechanisms, and that PDK4 enzyme activity is positively regulated by cellular ATP and NADH levels, which may be increased after PHx.

##### 3.3.1.2 Mitogenic signaling triggered by bile acids in the intestines through enterocytic farnesoid X receptor

Hepatectomized mice in which *eFxr* was genetically deleted exhibit a considerable reduction in ileal *Fgf15* transcript levels and increased hepatic transcript levels of downstream target *Cyp7a1* [254] (*Cyp7a1* is negatively regulated by hepatic FXR, section 4.1 and 4.2 and Table 1). These effects coincided with elevated plasma bile acid concentrations and stalled hepatocyte proliferation [254], the latter as a result of impaired cell cycle progression [257]. The deficiency in hepatocyte proliferation could be restored in *Fgf15*^−/−^ mice by adenoviral transduction of FGF15 [254], underpinning the involvement of intestinal FGF15 in liver regeneration. The results were reproduced in subsequent mouse PHx studies regarding ileal FGF15-mediated hepatocyte and cholangiocyte proliferation [22,257].

As mentioned earlier, activation of eFXR transcriptionally induces FGF15. Using primary hepatocytes, Holt et al. [277] showed that the promoter region of human *FGF19* contains an FXR-responsive element in isolated primary hepatocytes. Inagaki et al. [276] followed up with an investigation in mice, demonstrating that, in conjunction with RXR, murine FXR binds to the *Fgf15* IR1 motif, a conserved FXR binding site. FXR directly regulates *Fgf15* transcription, leading upregulation in intestinal epithelium following oral administration of the FXR agonists GW4064 and CA. Upon production of the functional protein, FGF15 is secreted basolaterally into the portal circulation [276,389] and subsequently binds to FGFR4/β-klotho receptor complex on the outer hepatocyte membrane [390-393] (Figure 6).

Mechanistically, FGFR4-induced mitogenic pathways have been investigated in only a few studies in the context of post-PHx liver regeneration, although proliferative signaling by FGFR4 has been established outside of this context (e.g. [394]). Uriarte et al. [22] observed impaired hepatocyte proliferation in *Fgf15^−/−^* mice compared to wild type mice, which was associated with reduced *hFxr* (6-36 hours post-PHx) and *Foxm1b* transcript levels (44-48 hours post-PHx) as well as decreased levels of FOXM1B downstream gene targets, including *Cdc25b* (36-44 hours post-PHx),* Ccnd1* (codes for cyclin D1, 24 hours post-PHx), and *Pcna* (proliferating cell nuclear antigen, involved in DNA replication and repair and chromatin remodeling [ 395], 36-44 hours post-PHx) compared to wild type mice (Table 1). Interestingly, transcript levels of the complete hepatic mitogens *Hgf* (44 and 72 hours post-PHx), *Hbegf* (6-44 hours post-PHx), and* Areg* (gene that encodes amphiregulin, 24-36 hours post-PHx) (section 2.3) were upregulated in *Fgf15^−/−^* mice [22]. The authors attributed this counter-regulation to the concomitant induction of alternative pro-proliferation pathways (e.g., EGFR signaling) as a reason for the observed, although latent, liver regeneration in *Fgf15^−/−^* mice.

Kong et al. [257] observed similar effects in *Fgf15^−/−^* mice and identified additional pathways. *Fgf15^−/−^* mice exhibited reduced protein expression of the MAPKs JNK and p38 and c-Myc (although p-ERK protein levels were elevated compared to wild type controls), confirming earlier reports on FGF15-induced MAPK signaling [396]. The knock-out mice also had decreased protein levels of JAK1, JAK2, and STAT3, indicating that FGF15 modulates proliferation through the JAK/STAT3 pathway [397,398]. As FXR, STAT3 is a positive regulator of *Foxm1b* [289] *(*Table 1), and is apparently activated by FGFR4 to induce cell cycle activity through FOXM1B in an IL-6- and TNF-α-independent manner [23]. Interestingly, STAT3 downregulation in *Fgf15^−/−^* mice occurred at augmented plasma IL-6 levels (a STAT3 inducer [84,95,96]) as well as increased hepatocellular SOCS3 levels (a STAT3 inhibitor [399]). Lastly, nuclear p65 protein (RELA, a functional unit of NF-κB [400]) was elevated in wild type animals but absent in *Fgf15^−/−^* mice, demonstrating that FGF15 promotes hepatocyte proliferation through NF-κB signaling [87,88] following PHx.

Tissue-specific knock-down and knock-out studies have also been performed for the FGF15 receptor FGFR4 to examine the underlying signaling pathways. Padrissa-Altés et al. [23] employed liposome-delivered siRNA to knock down hepatic FGFR4 in mice and observed comparable gross effects as in the *Fgf15^−/−^* mice [22,257] after PHx. Hepatocytes in the FGFR4 knock-down group exhibited reduced and latent proliferation and, besides decreased expression of *Foxm1b* (48 hours post-PHx) and *Stat3* (24 hours post-PHx), decreased levels of *Ccna1* and *Ccnb1* (codes for cyclins A2 and B1, respectively, 48 hours post-PHx) were also reported. It should be noted that Padrissa-Altés et al. used liver-specific FGFR4 knock-down mice and that these results were not reproducible in *Fgfr4^-/-^* mice (which had genetically deleted FGFR4), which did not exhibit aberrant liver morphology or regeneration pattern [401].

Taken together, the studies suggest that at least 4 proliferative pathways are activated by eFXR-induced FGF15 in post-PHx liver regeneration (Figure 6 and Table 1):

1. FGF15/hFXR/FOXM1B → mitosis
2. FGF15/FGFR4/JAK/STAT3/FOXM1B → mitosis
3. FGF15/MAPK (JNK/p38/ERK?) → mitosis
4. FGF15/NF-κB → mitosis.

How FGFR4 is linked to FGF15 and STAT3-, MAPK-, and NF-κB signal transduction, and how FGF15 feeds (direct) signals into these pathways remains to be determined. Moreover, it is important to understand the influence of cytokines and growth factors on these pathways, as these play prominent roles in liver regeneration (section 2.2 and 2.3).

Several pertinent inflammatory stimuli such as LPS and IL-1β have been reported to inhibit hepatic β-klotho and FGFR4 expression in mice. In human cell lines (Huh-7 and HepG2), IL-1β suppressed β-klotho transcription in a JNK- and NF-κB-dependent manner and blocked FGF19-induced ERK1/2 activation and cell proliferation [151]. Finally, a recent study [402] using human liver tissue slices demonstrated a 20-fold transcriptional upregulation of FGF19 following incubation with obeticholic acid, a potent FXR agonist [ 403]. To date, FGF19 expression in hepatocytes following FXR stimulation has been quite elusive. These new data, however, suggest that hepatocellular FGF19 may also facilitate autocrine proliferative signaling in the early phase of liver regeneration after PHx.

As a side note, FXR-activated downstream cascades may also amplify liver regeneration by peripheral mechanisms. For example, the human complement C3 gene contains FXR response elements in the proximal promoter region [404]. Complement C3a is elevated after PHx in mice and humans [8-10], possibly in part by the perturbed bile acid homeostasis that ensues after liver surgery [24-26]. C3a activates innate immune cells such as Kupffer cells and neutrophils to stimulate the sterile immune response [8-10] that triggers liver regeneration via IL-6 and TNF-α [36,84,85]. Complement proteins are therefore auxiliary hepatomitogens [9] (section 2.4) induced in part by bile acid-FXR signaling after PHx.

##### 3.3.1.3 Mitogenic and metabolic signaling through hepatic farnesoid X receptor in humans

The promoter of the human *FGF19* gene contains an FXR response element [277]. Accordingly, upon FXR stimulation by obeticholic acid, transcriptional upregulation of FGF19 was seen in human enterocytes [402]. Therefore, it is likely that, activated human eFXR induces *FGF19* expression in enterocytes. It should be noted that, under cholestatic conditions, FGF19 expression in human hepatocytes has been reported [405].

FGF19 activates ERK1/2 through the FGFR4/ β-klotho receptor complex and subsequently induces hepatocyte proliferation, similar to FGF15-induced cell proliferation in rodents. Whereas β-klotho expression in mice is inhibited by LPS and IL-1β, human β-klotho expression is only affected by IL-1β but not by LPS [151].

Besides regulation of cell cycle progression, human hFXR has additional effects. Due to FXR response elements in the human complement C3 gene [404], complement C3a expression increases after PHx in hepatocytes, enhancing the effect of earlier induced cytokines. Also, FXR is essential for metabolic support of the liver after PHx. PDK4, an FXR target gene, regulates pyruvate metabolism and thus gluconeogenesis. Xie et al. [27] showed that FXR stimulation by both CDCA as well as GW4064 resulted in production of lactic acid, pyruvic acid, and glucose-6-phosphatase in human HepG2 cells. SiRNA knock-down inverted these processes. These results suggest that FXR is important for generation of glucose and amino acids and therefore essential for biomass generation.

#### 3.2.2 Retinoid X receptor signaling in liver regeneration

RXR is a nuclear receptor comprising α, β, and γ isoforms and is activated by mainly 9-cis retinoic acid. Given that RXR is often an obligated heterodimer partner for other nuclear receptors, loss of RXR is expected to affect various nuclear receptor signals in the liver [ 406]. The proliferative and cell cycle regulatory properties of RXR have been corroborated in vitro [407], in rodents treated with RXR agonists [408,409], and in rodents with genetically deleted *Rxr* [348]. Principally, pharmacological induction of RXR leads to hepatomegaly, whereas genetic ablation of *Rxr* leads to hepatocyte hypoproliferation.

In terms of post-PHx liver regeneration in mice, genetic deletion of *Rxra* (codes for RXR-α) translated to a 20-hour delay in hepatocyte proliferation that coincided with reduced transcript and protein levels of hepatomitogens (HGF, FGF2 [410], and PDGF) and latent onset of pro-proliferative signaling [347]. Similar results were obtained in lecithin:retinol acyltransferase-deficient (*Lrat^−/−^)* mice [411], which lack hepatic retinoid stores and hence possess minimal RXR activation potential. In contrast to the *Rxr^−/−^* genotype [347], *Lrat^−/−^* mice had decreased levels of *Tgfa* (codes for TGF-α, a regeneration-promoting growth factor, section 2.3) [411]. Also, the replication forks were stalled in the G1 phase in *Rxra^−/−^* mice as a result of compromised PPAR-α/ BMAL1/REV-ERB/p21 cell cycle regulation. This stalled cell cycle was characterized by decreased levels of cyclins A2, -B1, - D1, and -E1 as well as CDK1, -2, and -4 at 48 hours after PHx compared to wild type animals [347]. In mice, PPAR-α is an obligate binding partner of RXR during hepatocyte proliferation [412], so the effects of RXR deletion on PPAR-α signaling and associated cell cycle regulators are warranted. Although supplementation with the endogenous ligand retinoic acid only increased cyclin E1 levels in *Rxra^−/−^* mice, wild type mice fed retinoic acid showed RXR-α/RXR-β binding to *Cdk1*, *Cdk2*, Cdk6, and cyclin D genes [290], suggesting genetic and epigenetic control of liver regeneration by RXR when all results are considered. Moreover, PHx-induced liver regeneration in wild type mice was associated with RXR-β activation [290] and increased mitotic and proliferative activity via upregulation of cyclins D and E, *Cdc25b*, c-Myc/*c-Myc*, and *Foxm1b* [290,293] (Table 1), despite the fact that retinoic acid levels in the liver drop somewhat after PHx [411]. At this point, no data are available on the role of bile acids in RXR-induced liver regeneration after PHx.

#### 3.3.3 Pro-regenerative signaling by pregnane X receptor

PXR, also referred to as steroid and xenobiotic sensing nuclear receptor, has a plethora of endogenous and exogenous ligands, including bile acids [413]. As the name implies, the chief function of this receptor is to identify toxic substances and consequently upregulate the expression of proteins involved in their detoxification and clearance [302]. The detoxification machinery predominantly entails CYP3A4, which is upregulated by PXR in conjunction with RXR [291]. The potency with which bile acids activate PXR is DCA > LCA > CDCA [414] (Table 1). PXR activation leads to hepatocyte proliferation [295,298].

Dai et al. [349] reported a temporary delay of liver growth in hepatectomized *Pxr^−/−^* mice at 36 hours post-PHx and persistently slowed liver growth from post-PHx day 5 onward. This culminated in a 17% reduction in liver mass on post-PHx day 10, which was associated with significantly reduced hepatocyte proliferation and decreased STAT3 protein levels. Wild type livers exhibited transient steatosis post-PHx, which was absent in *Pxr^−/−^* mice. PXR is known to positively regulate hepatic lipogenesis via transcriptional induction of PPARγ and CD36 [300,301] (Table 1). Expression of genes that mediate lipid metabolism (*Ppar-α, Ppar-γ*, fatty acid translocase, acetyl-CoA-carboxylase 1, and long-chain free fatty acid elongase) was lower in *Pxr^−/−^* mice after PHx, indicating that impaired fatty acid uptake and lipogenesis may account for delayed liver regeneration in PXR-null mice. Furthermore, a recent liver regeneration study in mice suggested that PXR activation by pregnenolone 16α-carbonitrile accelerated cell cycle activity via inhibition of forkhead box O3 (FOXO3) [296], a transcription factor that negatively regulates cell growth, proliferation, and differentiation in the phosphoinositide 3-kinase (PI3K) pathway [415]. Presently, no data are available on the role of bile acids in PXR-induced liver regeneration after PHx. Nevertheless taken that PHx is followed by a transient bile acid overload [22,24-26] and PXR is activated by bile acids [414], enhanced PXR activity after PHx is not unlikely. However, PXR is under negative control of IL-6 [297] (Table 1), so the proliferative effects of activated PXR may be tuned down by certain cytokines. IL-6 itself is also a mitogen after PHx (section 2.4), and therefore the exact interplay remains to be investigated.

#### 3.3.4 Pro-regenerative signaling by constitutively active/androstane receptor

CAR is a xenobiotic and endobiotic sensor and therefore transcriptionally regulates genes including CYP2B, CYP2C, CYP3A, sulfotransferases, and glutathione-S-transferases, amongst others involved in detoxification and elimination of such compounds [305-308,314] (Table 1). A unique feature of CAR, as opposed to other nuclear receptors, is that the receptor shows basal activity in the absence of ligands in human hepatocytes, which can be enhanced by the binding of agonists, such as LCA. Following ligand binding, CAR migrates to the nucleus and binds to DNA as a monodimer or CAR/RXR heterodimer to activate transcription of target genes [416]. Mice treated with CAR agonists develop hepatomegaly [417,418], but *Car^−/−^* mice paradoxically exhibit only modestly impaired liver regeneration after PHx [28]. CAR also regulates the biogenesis of critical cell components in mice after PHx [419].

Several pathways lie at the basis of CAR-mediated proliferative signaling. One of the better studied pathways entails growth arrest and DNA-damage-inducible, beta (GADD45β), which is a transcription factor with pleiotropic functions that encompass cell proliferation [317], pro-inflammatory signaling [318], and positive transcriptional regulation of nuclear receptors (RXR, CAR, PPARs) [315,316] (Table 1), albeit strongly dependent on the cell type. In the murine liver, GADD45β is profoundly expressed during early compensatory regeneration [419]. Studies in wild type and *Tnfr^−/−^* mice demonstrated that CAR activation with the selective CAR agonist TCPOBOP induced GADD45β and cyclin D1 in a TNF(R)-independent manner, which was abolished in *Car^−/−^* mice [304]. Furthermore, TCPOBOP-primed mice with deleted *Gadd45b* (rodent gene for GADD45β) exhibited stalled liver regeneration and transcriptional repression of downstream CAR target genes after PHx, despite intact proliferative signaling [420]. This study further unveiled that both CAR and GADD45β bind the CAR regulatory element of the *Cyp2b10* gene, underpinning the signal-amplifying role of GADD45β in terms of CAR signaling [420] (Table 1).

Another CAR signal amplifier is steroid receptor co-activator-3 (SRC-3) [421], a transcriptional co-activator that assists nuclear receptors in the upregulation of gene expression [422]. *Src^−/−^* mice present with reduced liver hyperplasia and decreased c-Myc (section 2.2) and FOXM1B (section 3.1.1) expression upon CAR activation with TCPOBOP [421] (Table 1). The same applies to β-catenin that, when co-activated with CAR, induces hepatocyte proliferation and hepatomegaly in mice [423]. An additional proliferative trigger for CAR-controlled mitogenesis may be oxidative stress [424], which is induced in the early regeneration phase (section 2.1). Finally, the inhibition of the nuclear receptor hepatocyte nuclear factor 4 (HNF-4)α by TCPOBOP-activated CAR was shown to downregulate miR-122 and upregulate corresponding promitogenic target genes (the transcription factor E2f1 and its downstream target *c-Myc*) in the murine liver [322]. This is in agreement with the data presented in section 2.4, where miR-122 downregulation was associated with a post-PHx pro-regenerative response [200,201]. It should be noted, however, that HNF-4α levels remain relatively stable during the early phase of post-PHx regeneration, as measured in rats [180].

Unfortunately, all abovementioned studies were conducted in the absence of PHx and relied solely on CAR activation, usually by TCPOBOP, as a result of which no definitive conclusions can be drawn on the role of CAR in post-PHx liver regeneration. The only study that used PHx in combination with *Car^−/−^* mice, aside from Huang et al. [28], examined the role of type 1 deiodinase and thyroid hormone activity in liver regeneration [350]. This investigation demonstrated that reverse tri-iodothyronine (rT3) levels rise after PHx in wild type and *Car^−/−^* mice, which could be reversed in wild type animals but not *Car^−/−^* mice with phenobarbital, an indirect CAR agonist. Levels of T3, a thyroid hormone that acts as a hepatic (auxiliary) mitogen in the post-PHx liver regeneration setting [425, 426], remained unaltered independently of CAR. The activity and expression of type 1 deiodinase, the enzyme that catalyzes the production of the endocrinologically potent T3 as well as the endocrinologically less potent rT3 from thyroxine (T4), was reduced by PHx and increased by phenobarbital in a CAR-dependent manner. The same applied to promitogenic T3 target genes, which were repressed after PHx as well as rT3 infusion but reactivated by phenobarbital, altogether implicating CAR in hormonal regulation of liver regeneration in mice. To add to the regulatory complexity, the proliferative activity of T3 is enabled by β-catenin [ 427], which in turn potentiates the mitogenic signaling of CAR in mice [423] as described in the previous paragraph. Evidently, the thyroid hormone/hepatic nuclear receptor signaling axes are still to be fully unraveled. How bile acids fit into these signaling networks is most elusive in terms of post-PHx liver regeneration.

#### 3.3.5 Pro-regenerative signaling by suppression of liver X receptor

LXR exists as an α and β isoform [428] that forms obligate heterodimers with RXR upon ligand activation to regulate gene expression [ 429]. The cognate LXR ligands are the sterols 24(*S*),25-epoxycholesterol, 22(*R*)-hydroxycholesterol and 24(*S*)-hydroxycholesterol, but the receptor also binds several metabolized bile acid species, including hyodeoxycholic acid, taurohyodeoxycholic acid, and cholestenoic acid [319,320] (Table 1).

Although hepatectomized *Lxr*^−/−^ mice do not exhibit impaired hepatocyte proliferation and liver regeneration compared to wild type controls, LXR activation in wild type mice with the selective LXR agonist GW3965 (Table 1) reduced hepatic levels of pro-proliferative STAT3, the cell cycle regulators FOXM1B and CDC25B (section 3.3.1.1), and the tissue remodelling protein matrix metalloproteinase 9 (MMP-9) [239], indicating that LXR exerts proliferation-repressive effects. The cause for LXR inactivation after PHx is rooted in the considerable decrease in sterol-based LXR ligands, namely 24(*S*),25- epoxycholesterol, 24(*S*)-hydroxycholesterol, and 27-hydroxycholesterol (Table 1), which occurs in the early phase of post-PHx liver regeneration in mice [239].

How bile acids mediate post-PHx liver regeneration in terms of LXR signaling is unclear, although one study suggests that LXR downregulation in mice may be mediated by bile acid overload-induced overexpression of nuclear receptor-interacting protein 1 (NRIP1) [401]. NRIP1 is a nuclear receptor that represses the activation of transcription by LXRα/RXRα as well as PPAR-α/RXRα heterodimers through its interaction with LXRα and PPAR-α [430]. In a mouse model, NRIP1 is considerably induced by dietary CA [401], a bile acid that is substantially elevated after PHx during the early phase of liver regeneration [24]. Consequently, bile acid-induced overexpression of NRIP1 after PHx may contribute to suppression of LXR-mediated gene transcription and consequent promotion of hepatocyte proliferation and liver regeneration.

#### 3.3.6 Pro-regenerative signaling by non-farnesoid X receptor nuclear receptors in humans

Pascussi et al. [297] showed that IL-6 inhibits expression of *PXR* and* CAR* in primary human hepatocytes. However, in contrast to FXR signaling, there is hardly any information on the links between non-FXR nuclear receptors in humans and PHx. In few studies, human cell lines were used to investigate nuclear receptor activity. For example, Frank et al. [416] showed that, in contrast to other nuclear receptors, CAR is continuously expressed in the cytoplasm of human breast cancer cells. Upon ligand binding, CAR translocates to the nucleus and activates transcription of target genes. Osabe and Negishi [ 431] used hepatocellular carcinoma cells to study ERK1/2-CAR interaction. Their results show that the ERK1/2 protein prevents CAR phosphorylation and subsequently avoids nuclear translocation of CAR. Yamamoto and Negishi [316] also studied CAR in the context of cell cycle progression. When CAR is stimulated, *GADD45B* gene expression in HepG2 cells is decreased. As described in section 3.3.4, GADD45β is a transcription factor involved in cell cycle progression [317] and expression of several nuclear receptors [315,316] (Table 1).

### 3.4 Role of bile acid-activated TGR5 in post-PHx liver regeneration

In addition to intracellular nuclear receptors, bile acids also activate a cell surface G protein coupled receptor TGR5 [432]. Activation of TGR5 in most target cells results in increased intracellular cAMP and activation of protein kinase A (PKA). TGR5 is expressed in the brown adipose tissue, muscle and the intestine where activation of TGR5 by agonists promotes energy expenditure and glucose homeostasis [433,434]. TGR5 is highly expressed in macrophages and activation of TGR5 inhibits cytokine production and inflammation [435-437]. Pharmacological activation of TGR5 has been shown to decrease inflammation and prevent atherosclerosis in mice [435]. In the liver and biliary system, TGR5 is highly expressed in cholangiocytes, Kupffer cells, and gallbladder epithelial cells, but not hepatocytes [437-440]. Activation of TGR5 is known to decrease cytokine production and Kupffer cell activation to decrease hepatic inflammation in mice [436]. Compared to adipocytes, myocytes, and macrophages that are usually exposed to low levels of bile acids in the systemic circulation, cholangiocytes are routinely exposed to high levels of bile acids, and bile acid signaling through TGR5 in cholangiocytes may be more pathophysiologically relevant. So far, studies have shown that TGR5 mediates the proliferative and anti-apoptotic role of bile acids in cholestatic liver diseases [26,441], which may on one hand protect cholangiocytes against bile acid toxicity but on the other hand promote cholangiocarcimona development and progression. After PHx, TGR5 KO mice showed significantly impaired regeneration, with increased liver injury and inflammation. Such detrimental effects of TGR5 knockout could be attributed to cholestatic liver injury upon bile acid overload after PHx, suggesting an important role of TGR5 in regulating bile flow and cholangiocyte cell death [26]. In addition, TGR5 KO mice have been shown to have a more hydrophobic bile acid pool, which may further predispose TGR5 KO mice to bile acid-induced toxicity after PHx. The mechanisms by which alterations in bile acid composition in TGR5 KO mice are caused still have to be elucidated.

## 4. Containment of post-hepatectomy bile acid toxicity in hepatocytes

To protect the liver from bile acid-induced hepatotoxicity [223], bile acid-laden hepatocytes have several mechanisms in place to curtail prolonged exposure to supraphysiological intrahepatic levels of bile acids. These mechanisms, which are an integral part of hepatocyte function [1,442-444] in terms of phase I-III endobiotic metabolism, entail:

- chemically altering the composition of the bile acid pool towards less toxic (more hydrophilic) species (phases I and II, section 4.1);

- regulation of substrate (cholesterol) availability for *de* *novo* bile acid synthesis (section 4.2);

- hepatobiliary transport (phase III, section 4.3).

These processes work in concert to gradually remove excessive bile acids from hepatocytes after PHx once the mitogenic signals have been relayed.

### 4.1 Modulation of hepatocellular bile acid composition during liver regeneration

Due to the transient bile acid overload after PHx [25,26], several nuclear receptors are activated and engage phase I and II metabolic processes to detoxify bile acids by chemical modification. Phase I bile acid detoxification is mediated mainly by CYP3A4, which hydroxylates the bile acids to more hydrophilic entities that are subsequently readily eliminated by the liver [445-448]. CYP3A4 expression is regulated by FXR [449] (section 3.3.1), PXR [291,295,414, 450 ] (section 3.3.3), CAR [299,309-311] (section 3.3.4), and VDR [333,451]. The nuclear receptors are activated after PHx as described in the referenced sections. CYP3A11 is also induced by bile acids such as LCA [295,414, 452 ], while CYP3A4 is induced by CDCA [449] - effects that have been mirrored in obstructive cholestasis in mice and human livers [453,454] - as a feed-forward protective mechanism against bile acid-induced hepatotoxicity.

Phase II metabolic neutralization of bile acid toxicity occurs through primarily sulfation and glucuronidation at the sterol’s hydroxyl groups [455-457]. Sulfation is catalyzed by sulfotransferase (SULT)2A1 [458,459] that is under positive transcriptional control of PXR and CAR [312,358,460 - 463], although negative regulation of SULT2A1 by FXR has been reported in mice [459] (Table 1). Glucuronidation is restricted to LCA- and CDCA-derived 6α-hydroxylated bile acids [295,414] and is catalyzed by UDP-glucuronosyltransferase (UGT)2B4 and UGT2B7 [464,465]. Several UGT isoforms in the human liver are under control of PXR and CAR [ 466], whereby *UGT2B4* is positively regulated by FXR [280] and PPARα [332]. *UGT2B7* seems to be repressed by bile acid-activated FXR [281]. In transgenic mice, the human *UGT2B7* gene was suppressed by CAR, which was subsequently shown to proceed via CAR-mediated inhibition of HNF-4α binding to the *UGT2B7* promoter in HepG2 cells [313] (Table 1). Accordingly, CYP3A4 and UGT2B4 seem to contribute to hepatoprotection during post-PHx bile acid overload.

Some of the nuclear receptors that govern the detoxification processes also regulate the excretion of the biotransformed bile acids (transporters are discussed in detail in section 4.3). For example, CAR activation leads to the overexpression of MRP4 (basolateral export of sulfated sterols) [312] and MRP2 (canalicular export of glucuronidated bile acids [467]) in mice [299], while activated PXR upregulates the murine basolateral exporters MRP3 and MRP4 [299], which transport sulfate- and/or glucuronite-conjugated bile acids out of hepatocytes [312,338, 468 ]. The regulation of bile acid transporters by nuclear receptors is also illustrated in Figure 4 and Table 1.

Finally, bile acid composition in mice is regulated by LRH-1 [263,267], but this regulation is expected to offset the hepatoprotection conferred by the nuclear receptors and TGR5. LRH-1 is a nuclear receptor in the liver [258] and intestines [469] that regulates bile acid homeostasis [258,260,263-267,470,471] through CYP7A1 [260,263-266] and CYP8B1 [267] (Table 1). CYP7A1 is the rate-limiting enzyme that catalyzes hepatic bile acid synthesis using cholesterol as substrate [225, 472 - 474]. CYP8B1, abundantly expressed in hepatocytes [475], is also responsible for bile acid synthesis [225,472]. Murine LRH-1 is repressed by CDCA [258] but upregulated by TNF-α under cholestatic conditions [261] (Table 1). Accordingly, LRH-1 is expected to affect bile acid composition after PHx. Mice in which *Lrh1* has been deleted exhibit increased relative amounts of hydrophobic bile acid species at the expense of reduced CA and taurocholic acid (TCA) in the bile acid pool [267]. In light of the transient bile acid overload after PHx, LRH-1 levels are expected to drop because of augmented FXR and small heterodimer partner (SHP) activation by bile acids (next section). The subsequent SHP-induced repression of LRH-1 [259,260] may therefore lead to a shift in bile acids to more hydrophobic species and skew the beneficial effects imparted by nuclear receptors and TGR5. However, numerous bile acid exporters are also hyperactivated during bile acid overload, as explained in section 4.3, so saturated hepatocytes may not necessarily have to be afflicted by the LRH-1 downregulation and the more toxic bile acid pool. At this point the exact function of LRH-1 during post-PHx liver regeneration warrants further investigation, particularly in the context of bile acid composition and hepatotoxicity.

### 4.2 Regulation of bile acid synthesis during liver regeneration

PHx results in transient bile acid overload in hepatocytes [25,26] as well as extensive hepatocellular cholesterol accumulation [293, 476 ]. While bile acids are instrumental in proliferative signaling and liver regeneration (section 3), they are also toxic to hepatocytes. This biochemical ambiguity also applies to cholesterol, the metabolic substrate for bile acid production [477]. On the one hand, cholesterol is exacted by mitotic hepatocytes for biomass generation and sustenance of various metabolic functions [412, 478 ]. That mitotic hepatocytes increase their chromatin cholesterol levels in order to facilitate proliferation (8-16 hours after PHx) [ 479], which is why cholesterol metabolic pathways are hyperactivated after PHx [209,293,476, 480, 481]. On the other hand, high cholesterol levels feed into the production of (potentially toxic) bile acids in hepatocytes, especially since CYP7A1 (the protein product of the *CYP7A1*/ *Cyp7a1* gene) is strongly upregulated 24 hours after PHx [482]. CYP7A1 is the rate-limiting enzyme in the bile acid synthesis pathway, using cholesterol as substrate [225]. Its upregulation in combination with elevated cholesterol levels [293, 476] may therefore account for the temporarily increased bile acid pool early after PHx.

#### 4.2.1 Increased hepatocellular cholesterol levels after partial hepatectomy

Hepatocytes control intracellular levels of cholesterol via synthesis and non-synthesis routes. The non-synthesis routes pertain to cholesterol import into and export out of hepatocytes. A study in rats found that the binding and uptake of cholesterol-enriched lipoproteins by hepatocytes isolated from the remnant liver was decreased early (16 hours) after PHx and restored to baseline levels in later phases of regeneration [483], suggesting that cholesterol uptake from the systemic circulation does not lie at the basis of post-PHx cholesterol loading in the early stages. However, there are at least 3 metabolic switches manifested at the level of synthesis and export during post-PHx liver regeneration.

In terms of the first metabolic switch, the increase in hepatocellular cholesterol is mediated by suppression of LXR signaling after PHx in mice [293]. As addressed in section 3.3.5, LXR is inactivated after PHx in mice, associated with the promotion of liver regeneration. One potentially contributing factor to the inactivation of LXR is upregulation of NRIP1 [401] (section 3.3.5), while another contributing factor is the substantial post-PHx reduction in LXR-activating ligands in hepatocytes [293]. The inactivation of LXR leads to increased cholesterol levels because LXR is a master regulator of cholesterol catabolism [484] and an inhibitor of cholesterol synthesis [485].

Second, intracellular cholesterol levels are coordinated by LRH-1, which positively modulates sterols through ABCG5 and ABCG8 [269]. Importantly, LXR also positively regulates the expression of *Abcg5* and *Abcg8* in mice [321] (Table 1). These ATP binding cassette half-transporters are replete in the liver and small intestine [244,486,487] and jointly transport cholesterol across the hepatocellular cell membrane into the canalicular system [319,321,488,489]. Indeed, ABCG5/ABCG8 deficiency in mice leads to augmented plasma sitosterol (a plant-derived cholesterol analogue) concentration and decreased cholesterol levels in bile [490]. Analogously, ABCG5/ABCG8 overexpression increases biliary cholesterol secretion and decreases cholesterol absorption [491]. Given that LRH-1 is negatively regulated by bile acid-activated FXR/SHP signaling [260,266] (Table 1), bile acids (e.g., DCA) may downregulate *Abcg5* and *Abcg8* [269] through FXR/SHP/LRH-1 during bile acid overload, a process that is aided by the concomitant downregulation of LXR in mice [293]. Although no information is available on LHR-1 in connection with *ABCG5* and *ABCG8* during post-PHx liver regeneration, it is anticipated that *ABCG5* and *ABCG8* are downregulated via bile acid-mediated suppression of LRH-1 signaling in early phases. Accordingly, Lo Sasso et al. demonstrated that *Abcg5* and *Abcg8* levels were dramatically decreased in hepatectomized mice [293], although in that study the repression was ascribed to LXR.

The third, albeit converse metabolic switch, is that hepatocytes reduce their cholesterol synthesis rate in the early phase of post-PHx liver regeneration (first 24 hours) [481,492]. Evidently, the net effect of the rate reduction is in itself not sizeable enough to decrease the hepatocellular cholesterol concentration in the very short term inasmuch as cholesterol levels peak at 1-3 days after PHx (in mice) [293]. However, in the longer term (72 hours after PHx and beyond) the cholesterol concentrations decline [293], as do intrahepatic bile salt levels [24].

In summary, the three metabolic switches after PHx are:

-ligand ↓ / NRIP1 ↑ → LXR ↓ → cholesterol synthesis ↑ / cholesterol catabolism ↓;

-bile acids ↑ → FXR ↑ → SHP ↑ → LXR ↓ / LRH-1 ↓ → ABCG5 ↓ / ABCG8 ↓ → cholesterol export;

-cholesterol synthesis ↓.

In light of these pathways, it is clear that hepatocytes regulate bile acid synthesis by mainly enzymological means (i.e., regulation of CYP7A1, next section) and transport (section 4.3) rather than through substrate control.

#### 4.2.2 Inhibition of bile acid synthesis during liver regeneration.

Bile acid production is susceptible to a negative feedback loop that that reduces bile acid accumulation and decreases the bile acid pool size [260,265,266,493]. This is mainly achieved by transcriptional inhibition of *Cyp7a1/CYP7A1,* which encodes the rate-limiting enzyme in the neutral bile acid synthesis pathway [494,495]. As mentioned earlier, bile acid activation of hFXR induces SHP, which inhibits the transactivational activity of LRH-1 and HNF4-α that are key stimulators of *Cyp7a1/CYP7A1* transcription [259,260,266] (Table 1). In addition, elevated intestinal bile acid levels inhibit hepatic CYP7A1 via the FXR-FGF15/19 gut-to-liver signaling axis [266,276,277,496]. Accordingly, the post-PHx bile acid overload in the liver [25,26] coincides with pleiotropic transcriptional repression of *Cyp7a1* [28]. In vivo, intestinal FXR/FGF15/19 appears to be the dominant route by which bile acids repress CYP7A1 in comparison to the hepatic FXR/SHP/LRH-1 route. Using tissue-specific knockout mice, Kim et al. demonstrated that FXR activation in the intestines but not the liver is necessary for short-term downregulation of hepatocellular *Cyp7a1* in mice [496].

In addition, murine *Cyp7a1* downregulation is also controlled by PXR, another nuclear receptor that is activated by LCA [295]. Correspondingly, bile acid species that have been reported to induce *Cyp7a1* repression include CDCA [324,497], DCA [498,499], and LCA [295], which constitute physiological ligands of FXR and/or PXR (section 3.3).

The bile acid response element of *CYP7A1/Cyp7a1* [323,324] is also targeted by HNF-4α, a nuclear receptor involved in lipid and glucose metabolism [328,329] (Table 1). CDCA can decrease HNF-4α-regulated downstream genes [325,326,500 ], indicating that this nuclear factor is involved in bile acid-mediated repression of CYP7A1. Despite a single study reporting unperturbed HNF-4α levels in the early post-PHx regeneration phase [180], mice in which *Hnf4a* (encodes HNF-4α) was genetically deleted exhibit derailed bile acid homeostasis [328,329]. HNF-4α further regulates *CYP8B1/Cyp8b1* and *CYP27A1/Cyp27a1*, that encode the acidic pathway regulating enzyme CYP27A1, via a HNF-4α binding site in the promotor region [325-327]. The promotor region of *CYP8B1* also contains a binding site for LRH-1 [268], which is increased on day 2-3 after PHx in mice [482]. Elevated levels of LRH-1 are therefore expected to augment CYP8B1 expression and promote bile acid synthesis. However, this signaling axis may be offset by negative *CYP8B1* regulation by FXR/SHP-dependent and independent pathways [501]. In vivo, HNF-4α and LRH-1 facilitate SHP binding to the *Cyp7a1* promoter and drive FGF15-mediated repression of bile acid synthesis [471]. Taken together, bile acid synthesis is suppressed during the early phase of post-PHx liver regeneration (Table 1).

Other negative mediators of *Cyp7a1* include NRF2 [331] (Table 1) that is overexpressed during the early phase of liver regeneration following PHx in mice [71-73,82] (section 2.1) and in part activated by certain bile acids such as UDCA [502]. Cytokines such as TNF-α [498] and IL-1β [497] (section 2.2) downmodulate *Cyp7a1* in rats through pathways involving FAS receptor/JNK/c-Jun [498,499] and HNF-4α [324,497]. Additionally, the growth factors TGF-β1 [ 503] and HGF [ 504] (section 2.3) downregulate *CYP7A1/Cyp7a1* through signaling cascades involving HNF-4α (induced by TGF-β1) [503] and protein kinase C (PKC)/ERK1 and 2/JNK (induced by HGF) [504], at least in human hepatocytes [505].

### 4.3 Regulation of bile acid transport during liver regeneration

To further deter the toxicity of bile acids [223], overloaded hepatocytes instate protective routes to limit bile acid influx at the basolateral end and eliminate excessive bile acids from the intracellular milieu via basolateral and canalicular exporters [355,506]. This process is also referred to as phase III metabolism.

#### 4.3.1 Regulation of basolateral bile acid import

As described before, the extraction of bile acids from the portal circulation by hepatocytes is facilitated by NTCP (encoded by *SLC10A1/Slc10a1*)*,* and OATP isoforms OATP1B1 (*SLCO1B1*), OATP1B3 (*SLCO1B3*), and OATP2B1 (*SLCO2B1*) [507-510], all located at the basolateral membrane of hepatocytes [238,509,510]. Studies in rats have shown that the expression of NTCP [506,511-513], OATP1, and OATP2 [506,512] considerably decline during post-PHx, early-phase liver regeneration (0.5-2 days). Additionally, expression of the specific isoforms OATP1B1 and OATP1B3 is decreased under cholestatic conditions [437,514]. Therefore, NTCP and OATP isoforms may prevent bile acid loading from the enterohepatic circulation. Accordingly, a significant rise in plasma bile acids has been observed in mice and rats within several hours after PHx [25,26]. Similar to regulation of CYP7A1, NTCP is transcriptionally downregulated by bile acids (Figure 4). The route by which bile acids suppress NTCP is multilayered and complex, involving FXR/SHP/LRH-1, HNF-1α, and HNF-4α [355,515]. In addition, NTCP is negatively regulated by TNF-α and IL-1β [ 516] but not NRF2 [331]. The same applies to OAT1B isoforms in terms of IL-1β [517] (Table 1.). It therefore seems that, during regeneration, the net expressional effect on these transporters is chiefly dictated by cytokine networks rather than metabolic networks (i.e., bile acid signaling).

#### 4.3.2 Regulation of basolateral bile acid export

Under normophysiological conditions, basolateral bile acid efflux is maintained at a very low rate, as most of the bile acids undergo canalicular secretion. Upregulation of basolateral bile acid efflux transporters is considered as an adaptive response to secrete bile acids into the systemic circulation for renal excretion. As is shown in Figure 4, the basolateral efflux of bile acids from hepatocytes is mediated by the transporters MRP3 (*ABCC3/* *Abcc3)* [518-520] and MRP4 (*ABCC4/Abcc4*) [468], both located at the basolateral membrane of hepatocytes [521,522] and both under positive control of e.g., NRF2 in mice [331] (section 2.1 and Table 1). Murine *Abcc3* is under negative control of IL-1β [516] and positive control of CAR [523] (section 3.3.4 and Table 1) and TNF-α via LRH-1 [261] (section 3.3 and Table 1), both implicated in post-PHx liver regeneration, albeit to different degrees. Following PHx in mice, MRP3 and MRP4 levels were shown to have increased up to 3.1-fold [524,525], which was associated with increased plasma bile acid levels [524]. Similar results were found for MRP4 in both mice and rats [273,513]. Since OATPs are bilateral exchangers, the previously mentioned A and B isoforms may also mediate basolateral efflux of bile acids [355], albeit to a limited extent because of their post-PHx downregulation [506].

The OSTα-OSTβ heterodimer is also located at the basolateral membrane of hepatocytes [278,526 ] and has been shown to transport bile acids [247] out of bile acid-overloaded hepatocytes [278]. As in the intestines (section 3.1.1), OSTα and OSTβ are under positive transcriptional control by several bile acid-activated nuclear receptors, including FXR and LRH-1 [262,273,278] (section 3.3 and Figure 4). The transporter complex is further induced by nuclear receptor ligands that are not activated by bile acids but do play a role in post-PHx liver regeneration, including glucocorticoid receptor (GR) [526]. Hepatic GR levels exhibit a considerable increase during the first 24 hours after PHx in rats [527], suggesting concomitant upregulation of OSTα-OSTβ and facilitated export of (toxic) bile acids. Contrastingly, OSTα-OSTβ are repressed by SHP [262] and IL-1β in mice [516] (section 2.2). The positive and negative regulation notwithstanding, the involvement of OSTα-OSTβ in bile acid flux modulation under conditions of PHx remains to be proven experimentally. Based on our knowledge and expertise, OSTα-OSTβ are induced by FXR and bile acids, where LCA plays a marginal-to-no role in terms of liver regeneration. LRH likely maintains basal transcriptional activity of the OST isoforms, but does not play an important role in response to bile acids.

In sum, these basolateral transporters, or at least MRP3 and MRP4, ensure that bile acids are pumped out of the cell back into the systemic circulation. All the abovementioned basolateral transporters are capable of also removing sulfated and glucuronidated bile acids from hepatocytes [528], indicating that the transporters work in concert with the hepatocyte detoxification machinery (section 4.1) to remove (conjugated) bile acids. The conjugated bile acids can subsequently be removed from the circulation by renal clearance [529-531].

#### 4.3.3 Regulation of canalicular bile acid export

Expression of BSEP, an export pump that traffics bile acids from hepatocytes into the biliary tree, is positively regulated by FXR [256,532,533], which heterodimerizes with RXR to bind the promotor region of *ABCB11* [256], as well as by NRF2 [330,331] and LRH-1 [259] (Table 1). While one study reported only slightly increased levels of BSEP after PHx in rats [506], FXR and NRF2 are activated during post-PHx liver regeneration [71-73,82,254,534] and thus may promote canalicular export of bile acids via BSEP in bile acid-congested hepatocytes post-PHx [370,506,534,535]. In support of the BSEP-mediated canalicular export of bile acids, Monte et al. demonstrated that bile acid levels in bile rise during the first 1-3 days after PHx in rats, and subsequently decline to near-baseline levels during the subsequent 11-13 days [24,482]. Correspondingly, mice fed CA and UDCA exhibit increased BSEP expression and elevated bile acid levels in bile [535,536].

In rats, increased canalicular export of bile acids from bile acid-overloaded hepatocytes also applies to MRP2 [506]. The same effect of CA and UDCA feeding to mice has been observed for hepatocellular MRP2 expression as was reported for BSEP expression [535,536]. Although this transporter is under negative control of IL-1β [516] and only one study reported unaltered or slightly increased BSEP mRNA levels after PHx [506], *Abcc2* (MRP2) is under positive control of several nuclear receptors that are activated during post-PHx liver regeneration (section 3.1), including RXR [292], FXR [279], PXR [279], and CAR [279,537], as well as the bile acids that activate these receptors and/or *Abcc2* directly [279,535,538,539] (Table 1). It therefore stands to argue that MRP2 is upregulated during the bile acid overload following PHx and assists in canalicular export of (toxic) bile acids. Finally, PHx in rats results in a considerable (up to 20-fold) increase in *Mdr1* mRNA levels in the remnant liver [540,541], but the significance of this upregulation in the context of bile acid trafficking during post-PHx liver regeneration is presently elusive.

### 4.4 Containment of post-hepatectomy bile acid toxicity in human hepatocytes

The modulation of hepatocellular bile acid composition during liver regeneration occurs through various enzymes that mediate phase I and II metabolic processes [445-448]. Phase I, hydroxylation, is mediated CYP3A4 that is under positive control of both bile acids and FXR in human hepatocytes [449]. Phase II metabolic reactions include sulfation, mediated by SULT2A1 [458,459] isoforms, and glucuronidiation, mediated by the UGT isoforms UGT2B4 and UGT2B7 [464,465]. While FXR [280] and PPAR-α [332] positively regulate *UGT2B4* expression, FXR negatively regulates *UGT2B7* expression [281]. Inhibition of *UGT2B7* was also seen as a result of CAR-mediated HNF-4α inhibition in HepG2 cells [313] (Table 1). These results suggest that bile acid metabolism is tightly regulated after PHx in humans in order to protect the liver from the post-PHx relative bile-acid overload. Besides, *CYP3A4* and *CYP3A11*, *CYP7A14* (the gene that encodes the enzyme that regulates bile acid synthesis from cholesterol) also contains a bile acid response element in its promotor region, suggesting that human hepatocytes also regulate bile acid synthesis after PHx though a negative feedback loop.

Additionally, basolateral bile acid import and export are well-controlled after PHx in humans. Human bile acid transporters have been identified and classified [239,508,510]. However, the expression of these transporters in humans has only been investigated in the context of cholestasis [278,542], and expression levels have not yet been measured after PHx. Chen et al. [542] investigated the expression of hepatocyte transporters and nuclear receptors in children with biliary atresia, a disease that leads to cholestasis. In early-stage cholestasis, expression levels of *ABCB11* (BSEP), *ABCC2* (MRP2), and *SCLO*-isoforms (OATP) where increased, suggesting that in situations of increased relative exposure to bile acids, human hepatocytes downregulate bile acid resorption. In HepG2 cells, FXR/RXR heterodimers positively regulate *ABCB11* (BSEP) [256,533]. CDCA induces *ABCB11* expression through FXR, whilst LCA works as a CDCA antagonist and decreases BSEP expression [533]. As a result of FXR activation, expression levels of OSTα-OSTβ also increase due to cholestasis in humans [278]. Human OSTα-OSTβ are also under positive control of GR, which is important after PHx [526].

## 5. Concluding remarks

Liver regeneration following PHx is a highly complicated process involving multiple organs and several types of signaling networks. The metabolic pathways that revolve around bile acid signaling occupy an ancillary yet important part in post-PHx liver regeneration.

The metabolic cues for liver regeneration are given off by bile acids directly after PHx and are hence facilitated by the temporary bile acid overload that lasts for up to 48-72 hours. The bile acids induce hepatocyte proliferation and liver regeneration through several nuclear receptors, either by direct binding or by indirect activation. Inasmuch as bile acids are inherently toxic, the state of bile acid hypersaturation cannot be sustained by the remnant hepatocytes that, while proliferating, also have to properly execute all aspects of liver function. Consequently, once the proliferative signals have been relayed by the bile acids, hepatocytes activate a bile acid detoxification and elimination machinery in order to protect themselves. These processes, induced in part by bile acids and nuclear receptors, encompass phase I-III endobiotic metabolism that predominantly results in the excretion of (biotransformed) bile acids into the canalicular system.

The excreted bile acids subsequently translocate to the intestines, where they mount a secondary wave of proliferative signaling in the liver. This system is based on the enterocytic reabsorption of bile acids, activation of FXR and consequent production of FGF19/FGF15, its release into the enterohepatic circulation, and the basolateral binding of FGF19/FGF15 to its cognate receptor, a complex of FGFR4/FGFR4 and β-klotho, on hepatocytes.

## 6. Future implications

Further research on the abovementioned pathways may have beneficial implications for various liver diseases and surgical procedures such as PHx. This last chapter will highlight points of interest for further research.

Firstly, no data are available on the role of several nuclear receptors in liver regeneration after PHx. Additional research on the effects of RXR, PXR, CAR, and LRH-1 in PHx-induced liver regeneration could help with understanding the role of bile acids in liver regeneration. It was shown that CAR influences thyroid hormone signaling [28,350], but further research has to be done to find out how the thyroid hormone/hepatic nuclear receptor signaling axis works. The kinetics of LRH-1 still have to be investigated, especially in the context of bile acid composition and hepatotoxicity. How bile acids influence liver regeneration remains unclear for all nuclear receptors except for FXR.

Secondly, several implications have been made on how bile acid flux is modulated under conditions of PHx. The significance of bile acid transporters in liver regeneration such as NTCP, OATP isoforms, MRP-2, -3 and -4, OSTα-OSTβ, BSEP, and MDR-1 remains to be proven experimentally.

Thirdly, it remains unclear how FGFR4, the FGF15/ FGF19 receptor, feeds signals into STAT3-, MAPK-, and NF-κB pathways and how cytokines and growth factors fit into these pathways. Ligand binding to this receptor initiates proliferative signaling that has only been partially elucidated in terms of mechanisms. This aspect of liver regeneration therefore warrants further research, especially since several FXR agonists have recently become available that promote liver regeneration via the gut-liver axis. Since cytokines and growth factors play prominent roles in liver regeneration (section 2.2), it is important to elucidate these pathways.

Finally, it was shown that downregulation of PDK4, a protein that regulates pyruvate metabolism, promotes liver regeneration [543]. Another study reported upregulation of PDK4 as a result of bile acid induced FXR activation [27], a mechanism that is in line with the anabolic demand during the liver regrowth face. It would be useful to further investigate the effects of PDK4 on liver regeneration in a PHx model since no study has shown that PDK4 enzyme activity is increased after PHx. Other signals that need further investigation entail the mechanism through which TGR5 confers biochemical protection during liver regeneration.

Both in vitro and in vivo studies are required for a better understanding of the importance of bile acids and their effects and effectors in the context of liver regeneration after PHx. Further elucidation of these pathways could be beneficial for patients with various liver diseases, including patients that have undergone a PHx.
